# Herbal Medicines in the Management of Diabetes Mellitus: Plants, Bioactive Compounds, and Mechanisms of Action

**DOI:** 10.3390/biom15121674

**Published:** 2025-12-01

**Authors:** Jamil Atef Chahrour, Zaher Abdel Baki, Dalia El Badan, Ghassan Nasser, Marc Maresca, Akram Hijazi

**Affiliations:** 1 Department of Biological Sciences, Faculty of Science, Beirut Arab University, P.O. Box 115020, Beirut 1107-2809, Lebanon; jamilchahrour@gmail.com (J.A.C.); d.badan@bau.edu.lb (D.E.B.); 2 Doctoral School of Science and Technology, Research Platform for Environmental Science (PRASE), Lebanese University, P.O. Box 115020, Beirut 1107-2809, Lebanon; ghassan.nasser@liu.edu.lb; 3 College of Engineering and Technology, American University of the Middle East, Egaila 54200, Kuwait; zaher.abdelbaki@aum.edu.kw; 4 Botany and Microbiology Department, Faculty of Science, Alexandria University, Alexandria 21568, Egypt; 5 Department of Chemistry, Lebanese International University-Beirut (LIU), Salim Salam Street, Mazraa, Beirut 146404, Lebanon; 6 Aix Marseille Univ, CNRS, Centrale Marseille, iSM2, 13013 Marseille, France

**Keywords:** diabetes mellitus, herbal-based medicine, hyperglycemia, medicinal plants, phytochemicals, antidiabetic effects, glucose transporter type 4 (GLUT4)

## Abstract

In recent years, herbal medicines (HMs) have been gaining significant attention as alternative or complementary therapeutic options. This is because synthetic drugs are expensive and have side effects, but also because herbal medicines have a rich content of effective bioactive compounds. These natural agents have been widely investigated for their potential in the prevention and management of chronic diseases including cardiovascular disorders, infections, metabolic disorders, neurological disorders, inflammatory disorders digestive diseases, oxidative stress-related diseases, and diabetes mellitus. In this review, we highlight the roles and impacts of various medicinal plants originating from diverse families, showing their bioactive characteristics, and the mechanisms through which they exert antidiabetic effects by regulating insulin secretion, oxidative stress, glucose uptake, and inflammatory pathways. In contrast to previous reviews, our study highlights the role of plants that are less explored, and integrates recent findings as well as future directions and biotechnological applications in plant-based management of diabetes.

## 1. Introduction

Diabetes mellitus (DM) is a chronic metabolic disorder characterized by persistent hyperglycemia that results from impaired insulin secretion, insulin action, or both. This disturbance affects carbohydrate, lipid, and protein metabolism, thus leading to a progressive metabolic dysfunction [1]. The global prevalence of diabetes has risen sharply, increasing from around 200 million cases in 1990 to nearly 830 million in 2022, with an estimated 14% of adults aged 18 years or more living with the disease. Additionally, in the year 2021 alone, diabetes was recognized to be a direct cause of approximatively 1.6 million deaths, half of them occurring before the age of 70. Furthermore, around 530,000 deaths from kidney diseases were found to be linked directly to diabetes disorder, and elevated blood glucose contributed to nearly 11% of all cardiovascular-induced deaths [2]. Regarding the clinical manifestations of diabetes, these can vary within individuals, depending principally on blood glucose levels and common symptoms, including: (i) polyuria, (ii) polydipsia, (iii) polyphagia, (iv) unexplained weight loss, (v) recurrent skin infections, (vi) genital infections, (vii) irritability, (viii) ketonuria, (ix) dysuria, as well as (x) blurred vision [3]. If these symptoms are left uncontrolled and/or untreated, diabetes progresses to more severe complications including cardiovascular disease, neuropathy, nephropathy, retinopathy, and chronic ulceration [4].

Type 1 diabetes (T1DM) results from autoimmune destruction of pancreatic β-cells, whereas type 2 diabetes (T2DM) is primarily driven by insulin resistance and impaired insulin receptor signaling [5,6]. Other forms of diabetes include gestational diabetes, monogenic diabetes, and secondary diabetes caused by pancreatic or endocrine disorders [4,7]. Several risk factors contribute to diabetes onset, including obesity, inflammation, genetic predisposition, aging, physical inactivity, and oxidative stress [8,9]. While conventional antidiabetic therapies such as metformin, sulfonylureas, insulin, GLP-1 receptor agonists, and SGLT-2 inhibitors have been reported to be effective in managing diabetes, they are unfortunately associated with several limitations and health-related side effects [10]. Such adverse effects include hypoglycemia, weight gain, gastrointestinal disturbances and, in some cases, cardiovascular risks [10]. Some of these effects often require a combination therapy in advanced T2DM, leading thus to increased healthcare costs and associated high risks of drug interaction [10]. Besides these critical health impacts, it should also be mentioned that the global economic burden of diabetes is shocking, with annual direct medical costs exceeding USD 727 billion worldwide, excluding the indirect costs that are related to productivity loss and disability [3]. This economic impact, combined with the growing prevalence of diabetes, especially in developing nations with limited healthcare infrastructure, highlights the urgent need for accessible, affordable, and most importantly, effective therapeutic alternatives [3,10].

Herbal medicines have long been used in traditional medical systems to manage various diseases, and modern experimental studies support their potential, showing antiviral, anthelmintic, antibacterial, anti-allergic, anticancer, antimalarial, antidiabetic, antioxidant, and anti-inflammatory activities [11]. Plants produce a wide variety of secondary metabolites, which can be classified based on their chemical structure, biosynthetic pathway, or solubility. These compounds, including alkaloids, phenolics, flavonoids, tannins, terpenes, saponins, glycosides, and volatile oils, contribute significantly to the therapeutic properties of medicinal plants [11].

Alkaloids, nitrogen-containing compounds derived from amino acids, are widely distributed in higher plants and show diverse pharmacological properties such as anticancer, antimalarial, antihypertensive, and analgesic effects [11]. Phenolic compounds, including flavonoids and tannins, are key antioxidants that help to regulate oxidative stress and inflammations, which are central to diabetes pathophysiology [11]. Flavonoids, often glycosylated, and tannins, demonstrate anti-inflammatory, antimicrobial, and cytoprotective properties [11]. Terpenes and terpenoids, which are the largest class of secondary metabolites, are composed of isoprene units and contribute in a wide range of biological activities, including cytotoxic, antimicrobial, and anti-inflammatory effects. Saponins, glycosylated steroids or triterpenoids, exert immunomodulatory, antioxidant, and cardioprotective properties [11]. Essential oils, which are volatile mixtures of terpenes and aromatic compounds, contribute to antimicrobial, anti-inflammatory, and antioxidant activities [11].

Therefore, these diverse phytochemicals are responsible for the antidiabetic, antioxidant, and anti-inflammatory effects of medicinal plants, highlighting the importance of integrating phytochemical composition with pharmacological studies in the management of diabetes.

Several reviews have discussed antidiabetic plants, but most remain limited to individual species, single mechanisms, or narrow phytochemical classes, resulting in fragmented and incomplete understanding. Additionally, existing literature often lacks an integrated comparison of phytochemistry, molecular mechanisms, in vitro and in vivo evidences, dosage considerations, and potential clinical relevance. No recent review has showed multiple antidiabetic plants while linking their bioactive compounds to shared and distinct molecular pathways, including glucose homeostasis, oxidative stress, inflammation, and metabolic regulation. This gap highlights the need for an updated and comprehensive review that unifies current findings in a structured and comparative manner.

Therefore, this review aims to summarize the key medicinal plants with reported antidiabetic activity, their major bioactive molecules, and the mechanisms through which they contribute to the management of diabetes mellitus.

## 2. Materials and Methods

A comprehensive literature search was performed to identify the studies focused on the use of herbal medicines to manage diabetes mellitus. Databases including PubMed, Scopus, Web of Science, and Google Scholar were searched by using combinations of keywords such as “herbal medicine”, “plant extracts”, “bioactive compounds”, “diabetes mellitus”, “type 2 diabetes”, and “mechanism of action”.

Articles were included if they:

(i) Contained the antidiabetic effects of herbal medicines or plant-derived bioactive compounds;
(ii) Provided insights for the mechanisms of action or therapeutic potential;
(iii) Were original research, reviews, or clinical studies published in English.

Studies were excluded if they were not related to diabetes, lack evidence for the antidiabetic activity, or were conference abstracts. The titles and abstracts were screened for relevance, followed by a full-text assessment to select studies that provided the most accurate and up-to-date information. This approach allowed for a comprehensive overview of the current knowledge on herbal therapies in diabetes mellitus management.

### 2.1. Biological Pathways Involved in Diabetes Management

Diabetes mellitus is a disorder that is characterized by impaired insulin secretion, insulin resistance, oxidative stress, and chronic inflammation, disrupting the balance of glucose in circulating blood and damaging various tissues. The mechanisms underlying these effects are well known, and herbal medicine has been shown to have an important effect in regulating blood glucose.

Insulin signaling and the incretin and oxidative stress pathways are considered the targets for herbal interventions. For example, some major plant compounds stimulate insulin signaling and promote glucose uptake, while other bioactive molecules modulate incretin hormones such as GLP-1, leading to improved insulin secretion and glycemic control [8].

Moreover, antioxidants that are derived from plants play a critical role by reducing reactive oxygen species (ROS) and suppressing pro-inflammatory cytokines such as TNF-α, IL-6, and IL-1β, therefore protecting pancreatic β-cells from apoptosis [10]. By targeting the multiple pathways that contribute in controlling diabetes mellitus, herbal medicine offers a multi-tasking approach that complements conventional therapies and highlights their therapeutic role in the management of diabetes [10].

**Figure 1:**
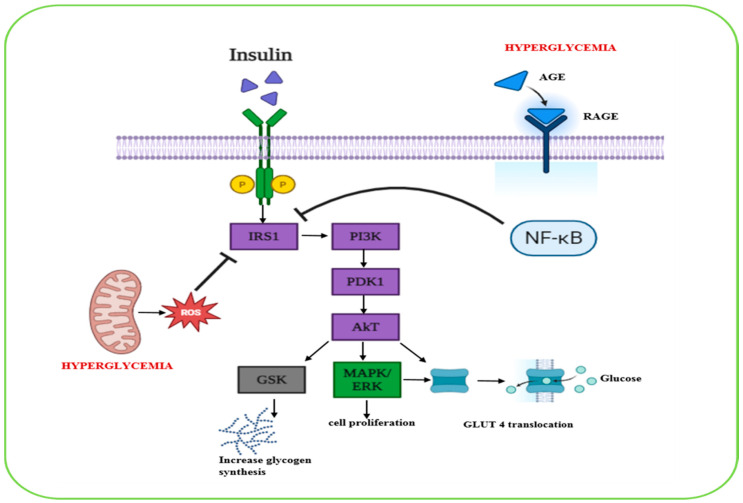
An illustration showcasing insulin binding to its receptor activates IRS1, which leads to subsequent Akt pathway activation. Activated Akt initiates three major cascades: 1—glycogen synthase kinase activation, 2—MAPK/ERK activation, 3—GLUT-4 translocation. Moreover, ROS and AGE inhibit IRS1 activation and disrupt insulin signaling.

Figure 1 shows these interconnected pathways, where medicinal plants might exert their effects in controlling glucose homeostasis.

### 2.2. Methodological Considerations and Quality Assessment

Research on herbal medicines involves several methodological challenges that need to be considered when interpreting their findings. The concentration of active compounds can vary significantly depending on the plant’s origin, growing conditions, harvest time, extraction procedure, and storage. Because different studies use different preparation protocols, comparing between the results becomes more difficult, and the reproducibility of findings is often limited. This problem is further amplified by the variability in clinical evidence, since studies remain small, short-term, or lack proper randomization. Although numerous in vitro and animal studies suggest promising antidiabetic effects, well-designed clinical trials, larger sample sizes, and long-term safety evaluations are still needed to confirm these benefits. More pharmacokinetic research is also required to clarify the absorption and metabolism of key phytochemicals. Along with these issues, ensuring the quality and consistency of plant material is essential in herbal medicine research. Differences in plant species, environmental conditions, and preparation methods can greatly affect chemical composition and therapeutic activity.

Modern quality-control approaches, such as chromatographic profiling and chemical fingerprinting, are used in an increased manner to verify extract identity, purity, and batch-to-batch consistency. These techniques help strengthen the reliability and reproducibility of studies on herbal interventions for various diseases including diabetes [12,13,14].

The rising global interest in medicinal plants further highlights the need for strong standardization practices. Herbal preparations appear in multiple forms (decoctions, powders, syrups, tablets), each influencing the chemical composition differently. Recent literature shows the importance of applying clear procedures for evaluating the plant materials, including microscopic, physical, chemical, and biological analyses, along with purity assessments and chromatographic techniques. These steps ensure that the herbal materials can meet the defined quality specifications and exhibit predictable biological effects [12].

This is considered particularly important since about 80% of the world’s population relies on medicinal plants for healthcare. However, many species still lack validated analytical methods, and some rely on low-selectivity tests that cannot guarantee the proper standardization. Importantly, chemical composition varies due to environmental and genetic factors, and for many plants, comprehensive phytochemical data remain incomplete. These limitations highlight the need for more rigorous analytical approaches in herbal standardization [13].

Techniques such as chemical fingerprinting have therefore become an important tool for assessing the quality of herbal raw materials and finished products [14]. It provides an overall chemical profile rather than relying on one or two markers, allowing researchers to classify the samples and link chemical patterns to biological activity using a chemometric tools. The proper standardization also requires evaluating the stability, efficacy, and safety of herbal products, as well as providing clear documentation for the appropriate use. Therefore, before herbal medicines can be reliably used in clinical settings, high-quality clinical trials and well-defined chemical specifications are required to ensure consistent therapeutic outcomes [14].

### 2.3. Medicinal Herbs: Past and Present Insights

**Figure 2:**
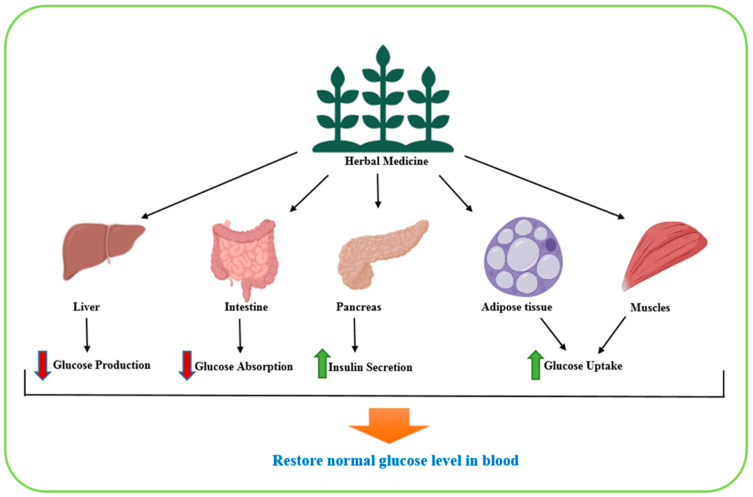
A graphical illustration showcasing the effect of herbal medicines on various organs during treatment against diabetes. Green and red arrows indicate increase and decrease, respectively.

In recent decades, complementary and alternative medicine, particularly herbal medicine (HM), has gained significant interest in the management of chronic diseases. Herbal preparations are applied across diverse cultures including China, Japan, New Zealand, Canada, the United States, and Russia due to their therapeutic value and traditional acceptance [15,16]. Plants contain diverse bioactive secondary metabolites, such as alkaloids, terpenes, phenolics, vitamins, and other nitrogenous compounds, which contribute to their defense mechanisms and other biological activities [17]. These phytochemicals have been shown to exert antimicrobial [18,19], antioxidant, and anti-inflammatory effects [20], in addition to displaying anti-carcinogenic properties, cardiovascular protection, neuroprotective activity, and regulation of apoptosis and cell cycle processes [21,22,23]. In the context of diabetes, numerous medicinal plants have demonstrated many hypoglycemic effects and now are used alongside conventional antidiabetic drugs (Figure 2) [24,25,26]. Their mechanisms include enhancing glucose uptake by adipose and muscle tissues, inhibiting glucose absorption from intestines and inhibiting glucose production from hepatocytes [24,25,26]. Importantly, plant-based therapies are generally considered safer, more affordable, and more accessible than synthetic drugs, and with fewer adverse effects [27].

### 2.4. Safety Considerations and Drug Interactions

While herbal medicines are generally considered safer alternatives to synthetic drugs, their use in diabetes management requires careful consideration of the potential adverse effects and drug interactions. Several of the medicinal plants discussed in this review exhibit strong hypoglycemic activity, which may enhance or overlap with the effects of conventional antidiabetic medications and potentially increase the risk of hypoglycemic episodes. For instance, bitter melon (*Momordica charantia*) and fenugreek (*Trigonella foenum-graecum*) have been reported to enhance insulin sensitivity and glucose uptake, which may necessitate dosage adjustments of the current insulin or sulfonylurea therapy [28]. Additionally, herbal medicines may affect the metabolism of conventional drugs through the modulation of various enzymes. For example, garlic (*Allium sativum*) and turmeric (*Curcuma longa*) have been shown to influence drug-metabolizing enzymes, potentially altering the pharmacokinetics of co-administered medications [28]. Healthcare professionals should be aware of these potential interactions and monitor patients closely when herbal medicines are used alongside the conventional therapies. Moreover, patient education regarding the importance and usage of herbal medicine by healthcare providers is essential for safer and more effective diabetes management [28,29].

### 2.5. Traditional Medicine and Diabetes

Before discussing each plant individually, it is important to mention that traditional medicine had been used for a long time in managing diabetes. For example, traditional medicine in China, India, the Middle East and other countries has used combinations of herbs and plant extracts to regulate diabetes by targeting multiple pathways that are involved in regulating blood glucose. While the composition, formulations, and specificity of plants differ between different regions, the main goal is to improve health and reduce the complications associated with diabetes mellitus. Highlighting these traditional approaches provides a better understanding of the wide variety of plants and bioactive molecules used in modern research, allowing the reader to better understand the historical usage of plants and recent scientific research in managing diabetes mellitus [30,31,32].

### 2.6. Antidiabetic Medicinal Plants

The 16 plants discussed in this section were selected because they are readily available and well-documented in the literature for their potential effectiveness in diabetes management.

#### 2.6.1. *Achyranthes aspera*

*Achyranthes aspera*, commonly known as Devil’s horsewhip, is a member of the Amaranthaceae family and is widely distributed as a weed throughout India [33]. Phytochemical screening has revealed that the plant contains a variety of bioactive compounds, including saponins, alkaloids, steroids, flavonoids, and terpenoids [34]. Traditionally, it has been used in Ayurvedic medicine for the treatment of conditions such as toothache, rheumatism, inflammation, bronchitis, microbial infections, skin diseases, colds, asthma, piles, rabies, and notably, diabetes [34,35]. Several studies have highlighted its antidiabetic potential. In an in vivo study, ethanolic seed extracts of *Achyranthes aspera* (300 mg/kg and 600 mg/kg body weight) were administered orally to streptozotocin (STZ)-induced diabetic rats for 28 days. The treatment significantly reduced blood glucose levels in comparison with the standard drug glibenclamide (5 mg/kg body weight) [35]. Although the findings are encouraging, the study relied on a single diabetic model and the sample size was small, which may restrict the strength of the conclusions.

Similarly, in vitro investigations showed that the leaf extracts of *Achyranthes aspera* inhibited the activities of α-amylase and α-glucosidase in a dose-dependent manner when compared to standard antidiabetic drugs [36]. The methanolic extract exhibited the strongest inhibition, reaching 55.0 ± 0.50% for α-amylase and 53.06 ± 0.23% for α-glucosidase at 160 µg/mL, whereas the petroleum ether extract showed 51.87 ± 0.00% and 46.0 ± 0.22% inhibition for the same enzymes at the same concentration. Because these enzymes play key roles in the carbohydrate digestion and the post-prandial rise in blood glucose, their inhibition represents a promising strategy for glycemic control [36]. These in vitro results are useful, but they do not provide information about how the compounds act in the body, so the real therapeutic relevance should be interpreted with caution.

Another study reported that the administration of *Achyranthes aspera* as herbal tea led to a significant reduction in blood glucose and serum triglyceride levels after one week of treatment [37]. The antidiabetic and hypolipidemic effects of the herbal tea were evaluated using five groups of rats: NC (non-diabetic rats given standard feed and tap water), DC (diabetic rats given feed and tap water), CON1 (diabetic rats given feed and 1 g/100 mL herbal tea), CON2 (diabetic rats given feed and 2 g/100 mL herbal tea), and CON3 (diabetic rats given feed and 3 g/100 mL herbal tea). The animals received either water or the herbal tea alongside their feed for 21 days. The fasting blood glucose levels were measured weekly, and *Achyranthes aspera* extracts showed a significant reduction in blood glucose concentrations compared with the control group [37]. The herbal tea showed beneficial effects. However, the study did not fully describe the composition or standardization of the preparation, which can influence reproducibility. Furthermore, experimental evidence in diabetic rats indicated that oral administration of *Achyranthes aspera* not only reduced hyperglycemia and dyslipidemia but also decreased oxidative stress and enhanced pancreatic insulin protein expression, confirming both its antidiabetic and antilipidemic activities [34]. Overall, the differences in extract type, dose, and treatment duration across studies indicate that more standardized experimental approaches are still needed.

#### 2.6.2. *Allium sativum*

*Allium sativum*, commonly known as garlic, is a flowering plant belonging to the family Amaryllidaceae and the genus *Allium* [38]. It is thought to have originated in Southern Europe and Central Asia and is now widely cultivated worldwide, with China being the largest producer. Garlic has been used in almost all major traditional medicinal systems, and it has been extensively used both as a culinary spice and a medicinal herb due to its broad spectrum of biological activities. Garlic possesses anticancer, antibacterial, antifungal, anti-inflammatory, and antioxidant properties. A study reported that insulin-resistant (IR) mice, induced by a high-oil, high-sugar diet followed by streptozotocin (STZ), were treated with GP at doses of 1.25, 2.5, and 5.0 g/kg·bw for five weeks. The fasting blood glucose level in the high-dose GP (DGH) group was approximately 42% lower than that of the diabetic model group, which demonstrates a notable hypoglycemic effect [39]. Although these findings are encouraging, the study used only a single animal model and did not provide details regarding sample size, which may limit the robustness of its conclusions. In addition, garlic has demonstrated beneficial effects in the management of several diseases such as cardiovascular disease, hypertension, and diabetes. Phytochemically, garlic is rich in a variety of plant material including fiber, adenosine sulfur, pectin, fructans, lectin, prostaglandins, nicotinic acid, carbohydrates, phospholipids, fatty acids, essential amino acids, selenium, potassium, iron, manganese, calcium, phosphorus, sodium, and vitamins C, E, B1, B2, and B6. Many of these constituents are believed to contribute to its therapeutic potential. In the context of diabetes, garlic has been shown to inhibit the enzyme α-glucosidase, a key enzyme in carbohydrate metabolism, thereby reducing subsequent blood glucose levels [40]. Clinical and experimental evidence also indicates that garlic lowers total cholesterol, triglycerides, and LDL cholesterol, while simultaneously improving insulin sensitivity [41]. Collectively, these effects support the use of *Allium sativum* as a complementary medicine in the management of diabetes and its associated metabolic complications.

#### 2.6.3. *Aloe vera*

*Aloe vera* has been used for centuries in cosmetics in multiple traditional medical systems and as well as modern healthcare products. It possesses a wide range of pharmacological properties, including anti-inflammatory, antioxidant, anti-tumor, laxative, wound healing, anti-aging, and antidiabetic activities [42,43]. Taxonomically, *Aloe vera* belongs to the family Liliaceae. Its bioactive compounds include minerals, lignin, enzymes, vitamins, sugars, amino acids, and salicylic acid [42,43]. Other key compounds such as alprogen and chromium have also been shown to lower blood glucose levels, restore pancreatic β-cell function, and enhance insulin activity [44]. Several studies have highlighted its role in diabetes management. For example, one study demonstrated that *Aloe vera* reduced blood glucose levels by inhibiting the glycation pathway and managing hyperlipidemia [42]. Another comparative study reported beneficial effects in both in vivo and in vitro models. In vivo, *Aloe vera* treatment (10 mL/kg, p.o.) increased serum insulin levels and reduced serum glucose concentrations when compared with control diabetic rats. In vitro experiments using isolated pancreatic islets from adult female albino rats further demonstrated that *Aloe vera* enhanced the insulin secretion from the isolated islets [45]. Still, the use of different experimental models and preparations makes it difficult to directly compare outcomes across different studies.

Moreover, in clinical trials involving patients aged 40–60 years, the administration of aloe leaf gel (300 mg capsules taken every 12 h for two months) alongside standard oral antihyperglycemic medications was evaluated in 35 individuals and compared with a placebo group. The findings demonstrated that *Aloe vera* gel improved glycemic control in patients with type 2 diabetes without producing significant changes in lipid profiles [46]. The results are promising, but the small number of participants and short intervention period mean that larger and longer trials are needed to confirm these clinical effects.

#### 2.6.4. *Amaranthus tricolor* (Lal Chaulai/Joseph’s Coat)

*Amaranthus tricolor*, also known as Lal Chaulai or Joseph’s coat, belongs to the family Amaranthaceae and is mainly distributed in temperate and tropical regions [33,47]. Traditionally, it has been used in multiple regions, especially Asia and Africa, to treat piles, leucorrhea, constipation, leprosy, and bronchitis [47]. Pharmacological studies have confirmed its anti-hyperlipidemic, anti-hyperglycemic, antiviral, anti-proliferative, cyclooxygenase enzyme inhibitory, antioxidant, and antidiabetic activities [33,48,49]. Experimental studies have demonstrated its potential against diabetes. For example, one study used *Rattus norvegicus* white rats that were assigned to five groups: a C(–) group, in which rats were neither exposed to a diabetogenic agent nor treated; a C(+) group as control; and T1, T2, and T3 groups, which were induced with MLD-STZ and then treated with red spinach (*Amaranthus tricolor* L.) extract at doses of 200 mg/kg bw, 300 mg/kg bw, and 400 mg/kg bw, respectively. Treatment with *Amaranthus tricolor* extract significantly lowered pancreatic malondialdehyde (MDA) levels and blood glucose concentrations in the STZ-induced diabetic rats, indicating a protective effect against oxidative stress [50]. The findings are encouraging, but the study relied on a single diabetic model and did not provide detailed sample-size justification.

Another in vitro study reported that the aqueous extracts prepared from the leaves and stems of two edible *Amaranthus* species, tested at concentrations of 0.25, 0.5, and 1 mg, exhibited notable antidiabetic activity, anti-lipase, anti-α-amylase, anti-α-glucosidase, and anti-acetylcholinesterase (AChE) activities, highlighting its therapeutic role in the prevention and management of diabetes [51]. Still, the in vitro enzyme inhibition does not account for metabolism or absorption, so these results should be interpreted with caution.

#### 2.6.5. *Anacardium occidentale* (Cashew Tree)

*Anacardium occidentale*, commonly known as the cashew tree, belongs to the family Anacardiaceae. Native to Brazil and now cultivated worldwide, its nuts are rich in bioactive compounds such as linoleic acid, oleic acid, tannins, tocopherols, flavonoids, anthocyanins, dietary fiber, folate, and unsaturated fatty acids (UFAs). Traditionally, it has been used in multiple regions to treat skin diseases, fever, pain, diarrhea, and arthritis [52,53]. Recent studies on the aerial parts of *Anacardium occidentale* have highlighted their strong antioxidant properties, which are closely linked to antidiabetic activity [54]. These extracts exhibit a potent free radical scavenging capacity while also enhancing the expression and activity of key endogenous antioxidant enzymes, including superoxide dismutase (SOD), catalase (CAT), and glutathione peroxidase (GPx). Such antioxidant protection is particularly relevant in type 2 diabetes mellitus (T2DM), where oxidative stress is a major driver of insulin resistance and β-cell dysfunction. The elevated reactive oxygen species (ROS) disrupt insulin receptor signaling and stimulate the release of pro-inflammatory cytokines, thereby worsening metabolic imbalance. By reducing oxidative stress, *Anacardium occidentale* extract supports insulin signaling, suppress inflammation, and protect pancreatic β-cells against apoptosis [54]. Scientific studies highlight its potential in diabetes management. For example, the inner bark extract of *Anacardium occidentale* significantly reduced hyperglycemia in alloxan-induced diabetic rats [55]. However, depending on an alloxan model limits the ability to predict long-term relevance, since alloxan causes acute β-cell destruction that does not fully mimic human type 2 diabetes.

Similarly, an animal study using rats divided the animals into four groups: group A (control), group B (given 200 mg/kg body weight of the extract orally), group C (fed an enriched fructose diet containing 25% fructose, w:w), and group D (fed the same fructose-enriched diet along with 200 mg/kg body weight of the extract orally). The treatments were administered for 21 days, and the methanolic stem bark extract was found to reduce hyperglycemia, hyperlipidemia, and lipid peroxidation, therefore offering protection against the diabetes-inducing effects of a high-fructose diet in diabetic rats [56]. While these results support the metabolic benefits, the study lacks details about the chemical characterization of the extract, making it difficult to identify the specific active constituents responsible for such effects. Moreover, leaf extract combined with Riboceine improved hepatic and renal function, restored β-cell activity, and modulated several diabetes-related genes, contributing to better glycemic control [57].

#### 2.6.6. *Annona squamosa* (Custard Apple/Sugar Apple)

*Annona squamosa*, a tropical tree belonging to the family Annonaceae, is traditionally used in multiple regions and known as custard apple or sugar apple. Various parts of the plant (roots, bark, seeds, leaves, and fruits) possess anti-ulcer, antiseptic, renoprotective, hepatoprotective, analgesic, antipyretic, anti-inflammatory, antioxidant, and antidiabetic properties. Consequently, it has been widely used in the treatment of gastritis, diarrhea, parasitic infections, rheumatism, splenic disorders, dysentery, and diabetes [58,59]. Experimental evidence supports its antidiabetic role. For example, in streptozotocin (STZ)-induced diabetic rats, fruit peel extracts of *Annona squamosa* were administered orally (250 mg/kg body weight) for 21 days and the effects were compared with glibenclamide. *Annona squamosa* significantly reduced blood glucose levels, lowered cholesterol, VLDL, LDL, triglycerides, and improved liver function markers [60]. Although the study showed clear beneficial effects, it relied on a single rodent model, which limits the applicability of the results. Another study was conducted in both rabbits (non-rodents) and rats (rodents), using the oral route of administration to evaluate the effects of *Annona squamosa* water extract in alloxan-induced diabetic rabbits (80 mg/kg bw) and STZ-induced diabetic rats (50 mg/kg bw). The findings showed that the extract effectively controlled hyperglycemia in both models by promoting glucose uptake in muscle and intestinal tissues and by enhancing insulin secretion from the pancreas [61]. These were promising results, but the use of different animal models and experimental designs between studies makes direct comparison more difficult. Furthermore, after oral administration of *Annona squamosa* aqueous extract to diabetic rats for 30 days, aqueous extract supplementation improved lipid metabolism, increased plasma insulin, and prevented diabetic complications in STZ-induced diabetic rats [62]. Still, the studies do not clearly show the extract standardization, which might affect reproducibility and identification of the active compounds responsible for the effects.

#### 2.6.7. *Berberis vulgaris* (Barberry)

*Berberis vulgaris*, commonly known as barberry, belongs to the family Berberidaceae. It is a deciduous, spiny shrub with elliptic leaves, traditionally used in multiple regions. Phytochemical analyses have revealed the presence of vitamins, proteins, lipids, tannins, carotenoids, anthocyanins, alkaloids, and phenolic compounds [63]. Among its bioactive compounds, the isoquinoline alkaloid berberine (a quaternary ammonium salt) is considered the most potent, with documented effects in preventing and treating several chronic diseases, including cardiovascular disorders, cancer, inflammatory diseases, depression, and diabetes [64]. Experimental studies support the antidiabetic effects of *Berberis vulgaris.* For example, in rats where diabetes was induced by intraperitoneal injection of STZ at a dose of 65 mg/kg bw with saponin extracts and 25 mg/kg aqueous extracts, significantly reduced blood glucose, cholesterol, and triglyceride levels were found compared with the control group, showing the potential of both aqueous and saponin extracts in managing STZ-induced diabetes rats [65]. Although the results are encouraging, the study relied on a single animal model, which may limit the broader applicability of the results.

In another study, a double-blind randomized clinical trial was conducted, in which 31 diabetic patients were randomly assigned to receive either 3 g/day of *Berberis vulgaris* fruit extract (BVFE) or a placebo for three months. Serum glucose, lipoproteins, apoB, apoA-I, insulin, homocysteine, and HbA1c levels were measured at baseline and at the end of the third month. The results showed that berberine prevented fructose-induced insulin resistance in rats by downregulating aldose reductase expression and inhibiting its enzymatic activity, therefore suppressing the polyol pathway that is overstimulated under hyperglycemia [66]. This clinical trial provides human evidence, but the small number of participants and short intervention period limit the external validity of such results.

The antidiabetic mechanisms of berberine (BBR) are multifactorial. It enhances glucose-stimulated insulin secretion (GSIS), improves insulin sensitivity, inhibits glucagon release, and stimulates pancreatic β-cell proliferation [63]. Berberine also promotes glucagon-like peptide-1 (GLP-1) secretion, modulates gut microbiota linked to type 2 diabetes, and reduces insulin resistance through upregulation of insulin receptor proteins (InsR) via the protein kinase C (PKC)-dependent pathway [63,64]. Furthermore, it exerts anti-inflammatory effects by reducing the expression of TNF-α, ROS, IL-6, and IL-1. On a molecular level, berberine also inhibits key enzymes such as α-glucosidase, protein tyrosine phosphatase 1B (PTP1B), and dipeptidyl peptidase-4 (DPP-4), leading to reduced intestinal glucose absorption. It also induces glycolysis by stimulating the translocation of glucose transporters GLUT1 and GLUT4 and by activating the AMP-activated protein kinase (AMPK) pathway [63,64,67].

#### 2.6.8. *Cinnamomum zeylanicum*

**Figure 3:**
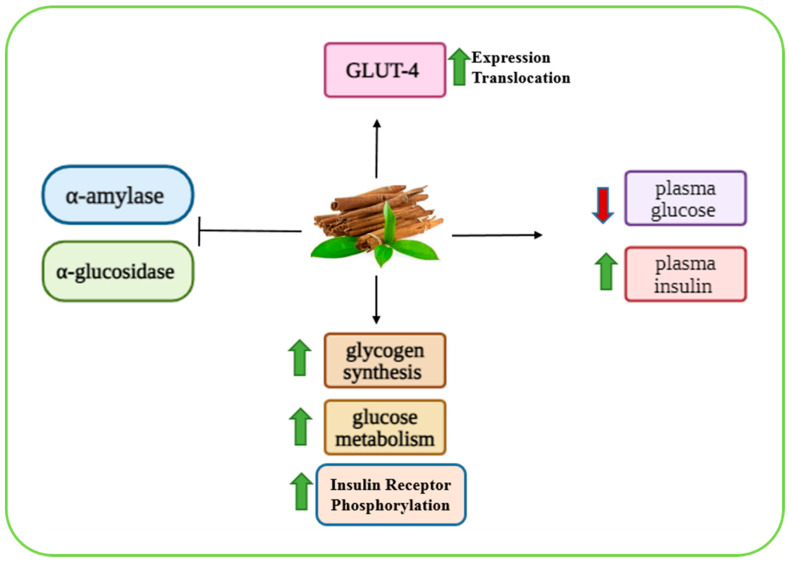
A graphical representation of the molecular mechanism of cinnamon inhibiting α-amylase, α-glucosidase, and plasma glucose, and stimulating plasma insulin, glycogen synthesis, glucose metabolism, and insulin receptor phosphorylation. Green and red arrows indicate increase and decrease, respectively. Image was created using BioRender.com.

*Cinnamomum zeylanicum*, also known as true cinnamon or Ceylon cinnamon, is one of the 250 species belonging to the genus *Cinnamomum* and family Lauraceae. Traditionally used in multiple regions as a spice and flavoring agent, it has also been widely recognized for its medicinal applications [68]. Phytochemical analyses have revealed that *Cinnamomum zeylanicum* contains numerous bioactive constituents with diverse biological activities, including anti-inflammatory, antimicrobial, antibacterial, anti-oxidant, anti-allergic, anti-pyretic, analgesic, and antidiabetic properties. The key components include eugenol (predominantly in the leaves), tannins, β-caryophyllene, linalool, cinnamic acid, weiterhin, mucilage, diterpenes, proanthocyanidins, cinnamaldehyde, cinnamyl acetate, cinnamyl alcohol, methyl chavicol, and methyl-hydroxy chalcone polymer (MHCP) [69]. In diabetic patients, Ceylon cinnamon has demonstrated multiple health benefits, such as lowering fasting blood glucose, reducing LDL cholesterol, increasing HDL cholesterol, and contributing to weight loss [70]. The antidiabetic mechanisms of *Cinnamomum zeylanicum* are multifaceted: (i) Inhibition of carbohydrate-digesting enzymes: cinnamon reduces intestinal glucose absorption by inhibiting pancreatic α-amylase and α-glucosidase [70,71,72]; (ii) regulation of glucose metabolism: It enhances glycogen synthesis and promotes glucose metabolism in peripheral tissues [70]; (iii) stimulation of glucose transporters: cinnamon upregulates GLUT-4 expression and facilitates its translocation to the plasma membrane, thereby increasing cellular glucose uptake [73]; and (iv) insulin-like activity. The extracts of *Cinnamomum zeylanicum* have been shown to decrease plasma glucose and increase plasma insulin in both animal (rats) and human models [72,74]. One active compound, cinnamtannin B1, exhibits insulin-mimetic activity by binding to the insulin receptors, triggering auto-phosphorylation, PI3K activation, and subsequent GLUT-4 translocation, which enhances glucose uptake [75]. Together, these findings suggest that *Cinnamomum zeylanicum* exerts its hypoglycemic effects through both insulin-sensitizing and insulin-mimetic actions, making it a valuable complementary therapy for diabetes management (Figure 3).

#### 2.6.9. *Curcuma longa* (Turmeric)

*Curcuma longa*, commonly known as turmeric, belongs to the family Zingiberaceae and is widely cultivated in Southeast Asia and traditionally used in various regions [76]. Type 2 diabetes mellitus (T2DM) development is strongly associated with oxidative stress, which activates protein kinase C and enhances polyol pathway flux [77]. Curcumin, the principal bioactive compound in *Curcuma longa*, exhibits strong protective effects on pancreatic islet cells through diverse molecular pathways. It promotes islet cell survival by attenuating reactive oxygen species (ROS) production and inhibiting pro-inflammatory mediators such as TNF and IL-1, IL-6, and IL-8 [76]. Curcuminoids enhance antioxidant defenses by regulating the expression of gamma-glutamyl-cysteine ligase, HO-1, and NAD(P)H:quinone oxidoreductase 1 at both the transcript and protein levels in human pancreatic islets [77]. This modulation results in increased antioxidant enzyme activity and elevated glutathione content, which collectively protect islet cells against oxidative damage. Moreover, curcumin interacts with a wide spectrum of signaling pathways, targeting growth factors, enzymes, transcription factors, cytokines, interleukins, and chemokines, underscoring its broad therapeutic value in diabetes management [76,78,79]. In patients with T2DM, treatment with *Curcuma longa* extracts has been shown to reduce fasting glucose levels and lower leptin, resistin, TNF-α, IL-6, and IL-1β while improving insulin resistance, hyperlipidemia, and hyperglycemia. Additionally, it increases adiponectin secretion and protects against pancreatic islet apoptosis and necrosis [77,80]. These effects confirm that curcumin, the major bioactive compound, plays a central role in diabetes prevention and treatment [80]. Several studies have supported these findings. A randomized, double-blind, placebo-controlled clinical trial demonstrated that curcumin supplementation significantly reduced the risk of developing T2DM in prediabetic individuals and improved β-cell function [81]. Another clinical study reported that in T2DM patients, *Curcuma longa* significantly decreased HbA1c, fasting blood glucose (FBG), and other metabolic parameters [82].

#### 2.6.10. *Gymnema sylvestre*

**Figure 4:**
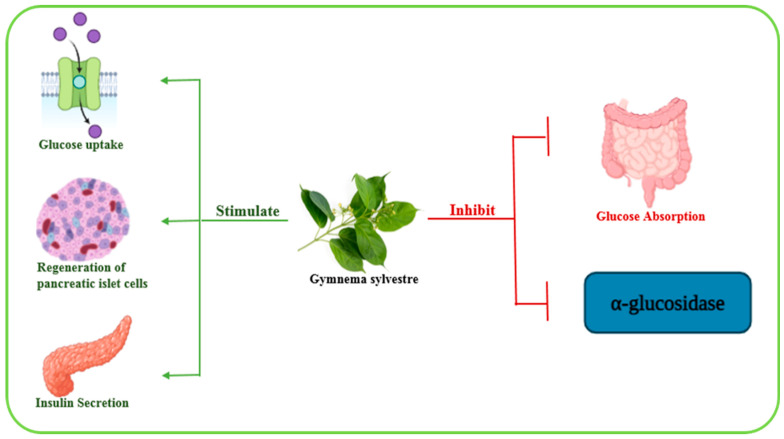
A graphical representation of a potential (and probable) molecular mechanism associated with the antidiabetic effect of the *Gymnema sylvestre* plant. Green and red lines indicate increase and decrease, respectively. Image was created using BioRender.com.

*Gymnema sylvestre*, also known as meshashringi or madhunashini, belongs to the Asclepiadaceae family and is primarily found in tropical Africa, Australia, and central and western India. Traditionally used across different regions, it has been used to treat inflammation, snakebites, asthma, eye disorders, and microbial infections, and also exhibits anti-obesity, anti-hypercholesterolemic, and hepatoprotective activities [83,84]. A study on 30 rabbits given *Gymnema sylvestre* alone and along with *Trigonella foenum-graecum* showed that the antidiabetic effects of *Gymnema sylvestre* were primarily related to the regeneration of pancreatic islet cells, increased insulin secretion, inhibition of intestinal glucose absorption, inhibition of α-glucosidase enzyme, and enhancement of peripheral glucose metabolism [85,86] (Figure 4). The leaves contain bioactive compounds including gymnemic acids, gurmarin, gymnemagenol, gymnomosides, and gymnemanol, which contribute to these effects [84].

More experimental studies have confirmed this hypoglycemic activity. For example, in a study on alloxan-induced hyperglycemic rats, *Gymnema sylvestre* was supplemented in the diet at doses of 250 mg/kg and 500 mg/kg bw. The pancreas and liver were collected for biochemical, gene expression, and histological analyses, and the results demonstrated that the leaf extracts of *Gymnema sylvestre* increased plasma insulin levels and significantly reduced blood glucose by modulating the expression of key genes involved in glucose metabolism and diabetes control, such as Irs1, Irs2, SREBP1c, Foxo1, Ins-1, Ins-2, and NF-κB [84]. Although the study demonstrates clear gene-level effects, it relied on a single animal model, which may limit the broader relevance. Another study demonstrated that rabbits treated with aqueous extracts of *Gymnema sylvestre* in combination with metformin, showed enhanced hypoglycemic effects, increased insulin secretion, and improved kidney and liver function markers (creatinine, serum urea, and liver enzymes) when compared to controls [87]. Nevertheless, the co-administration with another compound like metformin introduces a confounding factor, making it more difficult to isolate the independent effects of *Gymnema sylvestre*.

#### 2.6.11. *Gynostemma pentaphyllum*

**Figure 5:**
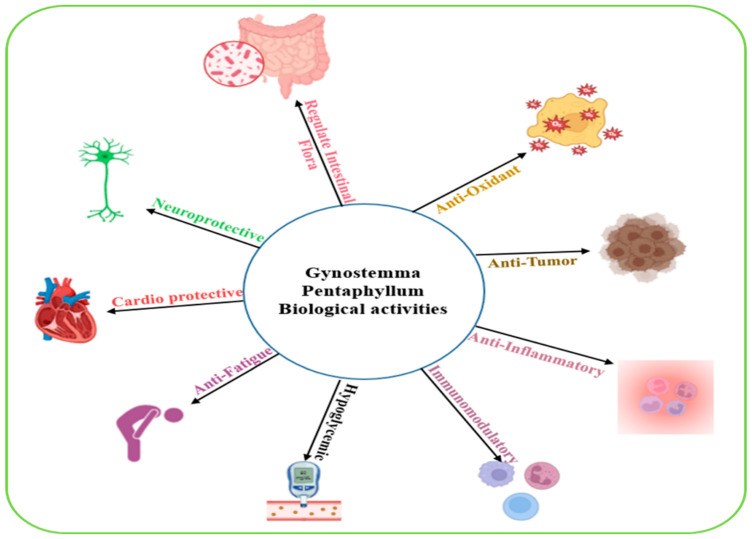
A schematic illustration summarizing the main GPS biological activities. Image was created using BioRender.com.

*Gynostemma pentaphyllum* is a climbing plant mainly found in the mountainous regions of Vietnam, Japan, North Korea, China, and Southeast Asia. It belongs to the family Cucurbitaceae and is traditionally used in various regions and especially Asia. The plant exhibits multiple biological activities (Figure 5), including anti-oxidant, anti-apoptotic, anti-hyperlipidemia, anticancer, anti-inflammatory, immunomodulatory, anti-fatigue, cardio protective, and neuroprotective, regulating micro flora and hypoglycemic effects [88,89]. Studies in patients with type 2 diabetes, in which participants received either 6 g/day of *Gynostemma pentaphyllum* (GP) tea or a placebo for four weeks, followed by a two-week washout period before crossover, demonstrated that GP significantly lowered plasma glucose when compared with placebo. Additionally, *Gynostemma pentaphyllum* was shown to improve insulin sensitivity and reduce hyperglycemia through the activation of the AMPK-mediated signaling pathway [90,91]. These are promising results, but the study included a relatively small number of participants, which may limit the broader applicability of the findings. Additionally, heat-processed extracts of *Gynostemma pentaphyllum* have been shown to increase GLUT4 expression and decrease liver histological damage in high-fat diet-induced glucose metabolic disorder models, indicating protection against metabolic and hepatic dysfunction [92].

#### 2.6.12. *Momordica charantia*

**Figure 6:**
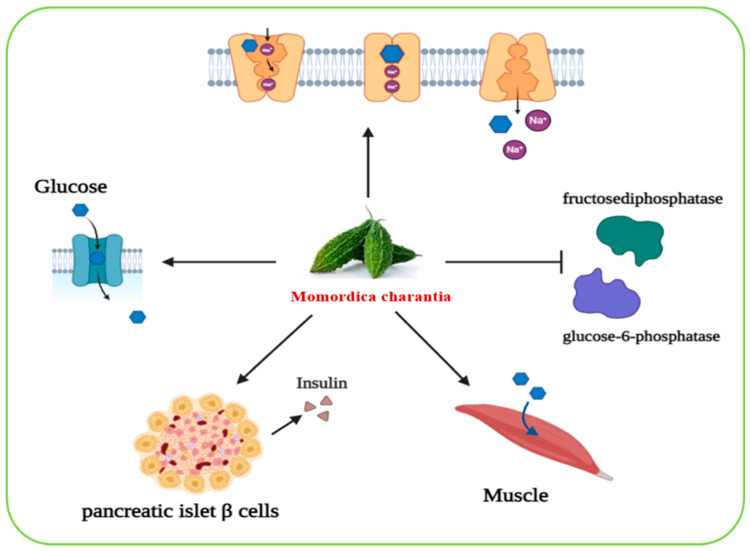
A graphical representation of the biological effects of *Momordica charantia* on glucose metabolism and insulin regulation. Image was created using BioRender.com.

*Momordica charantia*, commonly known as bitter gourd or bitter melon, is a tropical and subtropical plant that belongs to the Cucurbitaceae family. It is widely cultivated in South America, East Africa, Asia, and India, and used in multiple traditional medical systems [93]. *Momordica charantia* is considered a nutrient-dense plant that is rich in essential minerals such as potassium, calcium, zinc, magnesium, phosphorus, and iron, as well as antioxidants and vitamins including vitamin C, vitamin A, vitamin E, vitamin B9, and several B-complex vitamins (B1, B2, B3) [35]. In addition, phytochemical analyses have identified various bioactive compounds such as steroids, alkaloids, triterpenes, proteins, phenolics, and lipids, which are believed to contribute to its therapeutic activities [93,94,95]. The antidiabetic properties of *Momordica charantia* have been well-documented. Its hypoglycemic effect is mediated through several mechanisms: (i) *Momordica charantia* reduces the activity of key gluconeogenic enzymes, including glucose-6-phosphatase and fructose-1,6-bisphosphatase, thereby suppressing hepatic glucose production; (ii) it promotes glucose transport into skeletal muscle cells and increases the expression of intestinal Na^+^/glucose co-transporters, both of which contribute to lowering postprandial blood glucose levels; and (iii) experimental studies suggest that *Momordica charantia* helps preserve the morphology and function of pancreatic islet β-cells, which are responsible for insulin secretion. This protective effect supports improved insulin production and overall pancreatic health [95,96,97]. Collectively, these mechanisms highlight *Momordica charantia* as a promising antidiabetic medicinal plant with multiple actions, including enhancement of peripheral glucose utilization, reduction of hepatic glucose output, and preservation of β-cell integrity (Figure 6).

#### 2.6.13. *Nigella sativa* (Black Seed/Black Cumin)

**Figure 7:**
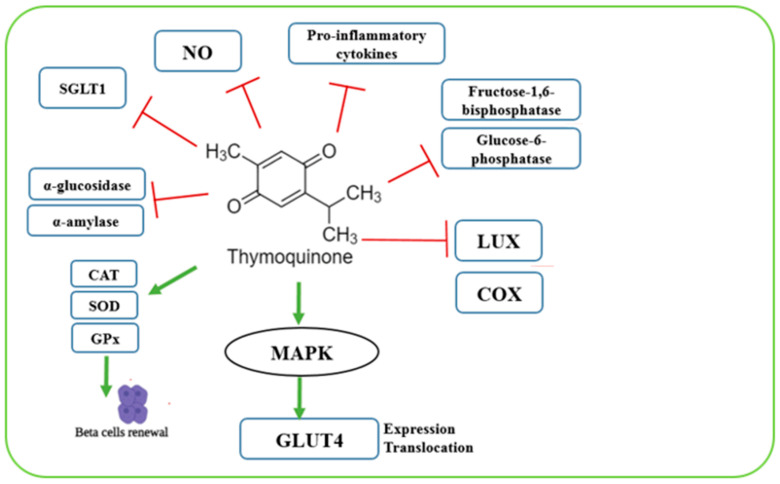
A graphical illustration of the antidiabetic actional mode of the nigella sativa plant. Green and red arrows indicate increase and decrease, respectively. Image was created using BioRender.com.

*Nigella sativa*, commonly known as black seed or black cumin, belongs to the genus *Nigella* L. in the family Ranunculaceae, order Ranunculales, class Magnoliopsida, division Tracheophyta, kingdom Plantae [98]. It is mainly distributed in Mediterranean countries and Iran. The oil derived from *Nigella sativa* contains bioactive compounds such as alkaloids, phenolics, and terpenes [99]. Traditionally, *Nigella sativa* has been used across several regions for the treatment of various chronic conditions including cancer, obesity, hypertension, and diabetes (Figure 7) [100]. The most important bioactive constituent is thymoquinone (TQ), which exhibits antidiabetic effects through multiple mechanisms. TQ inhibits α-glucosidase and α-amylase digestive enzymes, stimulates MAPK pathway activation that leads to an increase in muscle GLUT-4 levels, inhibits the activity of COX and lipoxygenase (LOX) enzymes, suppresses hepatic gluconeogenesis by targeting fructose-1,6-bisphosphatase and glucose-6-phosphatase, suppresses pro-inflammatory cytokines such as IL-6, TNF-α, IFN-γ and IL-1β, inhibits NO production, and reduces intestinal glucose absorption by inhibiting sodium-glucose linked transporter 1 (SLGT1) [98]. Additionally, since diabetes is associated with increased reactive oxygen species (ROS) that damage pancreatic β-cells, *Nigella sativa* enhances antioxidant defense by increasing the activity of superoxide dismutase (SOD), glutathione peroxidase (GPx), and catalase (CAT). It also protects and stimulates β-cell proliferation, decreases liver glucose production via gluconeogenesis, enhances insulin secretion, decreases insulin resistance, decreases fasting blood glucose, and produces a significant rise in serum insulin [98,101,102].

Several experimental and clinical studies have confirmed these effects. For instance, Type 1 diabetes mellitus was induced in 24 rats by a single intraperitoneal injection of streptozotocin (STZ) at 65 mg/kg. The animals were equally divided into four groups: (1) control group, (2) diabetic untreated group, and (3) and (4) groups treated with different doses of *Nigella sativa* oil (NSO) at 0.2 and 0.4 mL/kg, respectively, for 30 consecutive days. The results showed that administration of the low-dose NSO protected the pancreatic islets and increased serum insulin levels in the type 1 diabetes models [103]. Although the results show a protective effect, the study used a single animal model with a small sample size, which may limit the broader applicability of such findings. Moreover, in a clinical observation, a 2-year case study was carried out on a 72-year-old man with type-2 diabetes, stage 3–4 chronic kidney disease, and congestive heart failure. The daily intake of *Nigella sativa* tea reduced body weight and blood glucose levels in the patient [104]. However, this single-patient observation cannot provide generalizable evidence; thus, larger clinical studies are needed.

#### 2.6.14. *Ocimum sanctum*

*Ocimum sanctum*, also known as holy basil, belongs to the family Lamiaceae and is predominantly found in the South Asian region. Traditionally used in multiple medical systems, its leaves have been applied to manage a wide variety of conditions including chronic fever, helminthiasis, dyspepsia, skin disorders, bronchial asthma, dysentery, catarrhal bronchitis, hemorrhage, and hypoglycemia. The hypoglycemic effect of *Ocimum sanctum* is related primarily to its ability to lower blood glucose levels and stimulate insulin secretion from pancreatic β-cells [105]. Several experimental studies have confirmed the antidiabetic potential of *Ocimum sanctum*. For example, the ethanolic leaf extract of *Ocimum sanctum* was evaluated for its hypoglycemic effect in alloxan-induced diabetic rats in a study that included four groups of six rats each, with glibenclamide used as the standard drug. Ocimum sanctum demonstrated a significant hypoglycemic effect when compared to glibenclamide (a standard sulfonylurea antidiabetic drug). The proposed mechanism involved an increase in intracellular calcium levels within pancreatic β-cells, which in turn enhanced the insulin secretion [106]. Although the study shows promising results, the small sample size and single animal model may limit the broader applicability of the study. Another study reported that the leaf oil extract of *Ocimum sanctum* was administered to male Wistar rats divided into three groups (*n* = 7 per group) and fed daily with the fixed oil for three weeks. The treatment significantly reduced blood glucose and serum lipid levels, while simultaneously increasing serum insulin concentrations in streptozotocin (STZ)-induced diabetic rats [107]. Like the previous study, the small sample size and single animal model may limit the broader applicability of this study, but taken together, these findings suggest that *Ocimum sanctum* exerts its antidiabetic effects through the stimulation of insulin secretion, improvement of lipid profile, and reduction of hyperglycemia, highlighting its potential as a supportive herbal therapy for diabetes management.

#### 2.6.15. *Punica granatum* (Pomegranate)

*Punica granatum*, commonly known as pomegranate, is a medicinal and edible plant belonging to the family Punicaceae (sometimes classified under Lythraceae). It is widely distributed across the Mediterranean region, Southeast Asia, Africa, Europe, and the northwestern provinces of China. Pomegranate leaves are particularly rich in triterpenoids, phenolic compounds, and tannins, which contribute to its pharmacological activities [108,109]. Traditionally used in multiple medical systems, pomegranate has been prescribed for the prevention and treatment of various conditions including dental disorders, erectile dysfunction, cardiovascular disease, cancer, and diabetes. Its therapeutic value is largely related to its potent antioxidant capacity, particularly in scavenging the free radicals and reducing oxidative stress that are considered key factors in the pathogenesis of diabetes [108]. The antidiabetic mechanisms of *Punica granatum* include stimulating pancreatic β-cell secretion, enhancing insulin expression, and increasing glucose uptake by upregulating the mRNA expression of *IRS-1* and *Akt* genes [109]. These effects improve insulin signaling and glucose metabolism. Several experimental studies support its antidiabetic potential. For example, in type 2 diabetic rats, the rat model was orally administrated with polyphenols extract at doses of 50 and 100 mg/kg for 4 weeks. The flower polyphenol extracts improved blood antioxidant status, lipid profile, liver glycogen storage, and insulin sensitivity, resulting in reduced blood glucose levels [110]. Another study involved oral administration of fresh fruit juice (500 mg/kg body weight) and peel extract (500 mg/kg body weight) in sixty adult male albino rats. The treatment resulted in an increase in the catalase (CAT) enzyme activity, improved pancreatic islet health, and exhibited antioxidant and lipid-lowering effects in diabetic male albino rats [111]. As with the previous studies, the small sample size and single animal model may limit the broader applicability of the study.

Overall, Punica granatum exerts its antidiabetic effect by combining antioxidant activity, β-cell protection, enhancement of insulin signaling, and improvement of lipid metabolism, making it a promising natural therapeutic agent for diabetes management.

#### 2.6.16. *Trigonella foenum-graecum*

**Figure 8:**
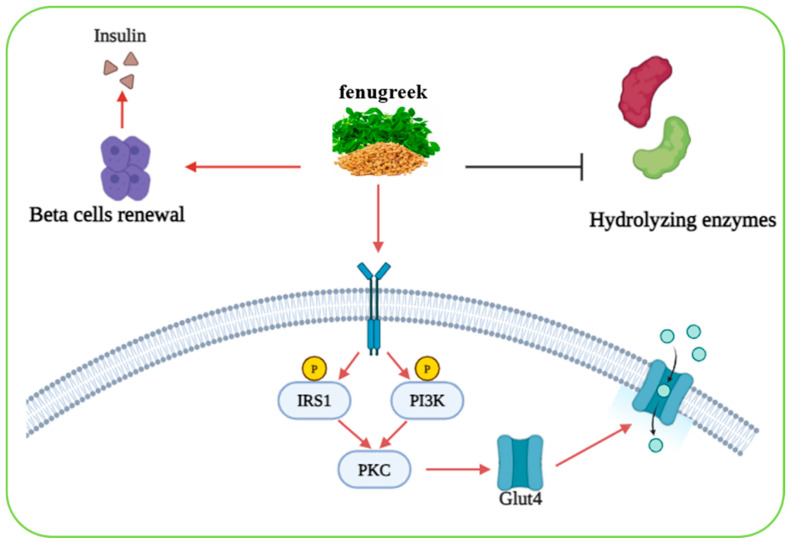
A schematic representation of the hypoglycemic effects of *Trigonella foenum-graecum* (also referred to as fenugreek). Image was created using BioRender.com.

*Trigonella foenum-graecum*, commonly known as fenugreek, is a medicinal plant belonging to the Fabaceae family traditionally used in multiple medical systems. Its leaves and seeds are nutrient-rich, containing essential vitamins, minerals, and macronutrients [112]. Fenugreek has long been recognized for its therapeutic properties, particularly as an antidiabetic herb, due to its diverse phytochemical composition. The seeds and leaves of fenugreek contain polyphenols, alkaloids, flavonoids, saponins, steroids, lipids, carbohydrates, hydrocarbons, galactomannan fiber, and amino acids [113,114]. Among these, diosgenin (a steroidal saponin) is considered the most bioactive compound, and is known to improve diabetic status by promoting pancreatic β-cell renewal and stimulating insulin secretion [114]. Another key compound, 4-hydroxyisoleucine, a plant-derived amino acid present in high amounts in fenugreek, has been shown to enhance insulin secretion and reduce insulin resistance in peripheral tissues such as muscle and liver [114,115]. The high fiber content of fenugreek seeds, particularly galactomannan, which constitutes 45–60% of the seed, contributes significantly to its antidiabetic effect. This soluble fiber forms a viscous gel in the intestine, thereby slowing glucose absorption and creating a physical barrier that reduces postprandial glucose spikes [114,115]. Additionally, fenugreek fibers inhibit lipid and carbohydrate-hydrolyzing enzymes, thereby further supporting glycemic control [115]. Experimental evidence also supports fenugreek’s role in modulating cellular glucose uptake. For example, in HepG2 cells, fenugreek treatment increased glucose uptake through upregulation of glucose transporter-2 (GLUT-2) mRNA levels [116]. Furthermore, studies in cell lines including CHO-HIRc-mycGLUT4eGFP, 3T3-L1-mycGLUT4, 3T3-L1, HepG2, and A431 demonstrated that fenugreek seeds enhanced tyrosine phosphorylation of IRS1 and PI3K proteins, leading to protein kinase C (PKC) activation. This signaling cascade facilitated the translocation of GLUT4 to the plasma membrane, thereby increasing glucose uptake from the blood [117]. Collectively, the diverse bioactive constituents of *Trigonella foenum-graecum* (fenugreek) exert significant hypoglycemic effects through multiple mechanisms. These include the regeneration of pancreatic β-cells, stimulation of insulin secretion, enhancement of insulin sensitivity, inhibition of carbohydrate-digesting enzymes such as α-amylase and α-glucosidase, and upregulation of glucose transporter activity. Furthermore, *Trigonella foenum-graecum* promotes insulin signaling by inducing tyrosine phosphorylation of insulin receptor substrate-1 (IRS-1) and phosphoinositide 3-kinase (PI3K), leading to the activation of protein kinase C (PKC) and subsequent translocation of glucose transporter type 4 (GLUT4) to the cell membrane, thereby facilitating glucose uptake [117]. These properties highlight *Trigonella foenum-graecum* as a promising complementary therapeutic agent for diabetes management (Figure 8).

## 3. Approaches for Enhancing for Bioavailability of Phytochemicals

Most of the bioactive molecules of medicinal plants, such as phenolics, glycosides, and flavonoids are water-soluble molecules, so that they have a low absorption and limited biological effects, in other words, low bioavailability [118]. This limitation in bioavailability is due to several factors, including large molecular size, multiple ring structures, and low lipid solubility that decreases the bioactive molecules’ intestinal diffusion and transport into the bloodstream [118]. In addition, many phytochemicals are bound tightly to the plant fibers or located within vacuoles, making them inaccessible during digestion [119].

To overcome these challenges, many strategies have been developed to improve and increase the bioavailability; for example, the use of solubility enhancers, making structural modification, or incorporation into lipophilic carriers [118]. Among the most promising technologies is using the phytosomes, so that natural molecules form complexes with phospholipids, leading to highly improved absorption of bioactive molecules through the gastrointestinal tract [118]. Phytosomes are safe and approved for usage in pharmaceutical and cosmetic domains, and have shown enhancement of bioavailability of hydrophilic flavonoids and other compounds when compared to conventional herbal extracts [118].

Moreover, delivery systems of molecules based on nanotechnology, such as polymeric micelles, liposomes, solid lipid nanoparticles, and nanogels, have been shown to be a powerful tool for increasing permeability and stability of phytochemicals [120,121]. These nanostructures encapsulate or bind bioactive compounds, protecting them from degradation and increasing their circulation time in the body [120,121].

For instance, Tsai et al. demonstrated that curcumin-loaded PLGA nanoparticle, significantly increases curcumin’s half-life [121]. Although niosomal formulations of *Gymnema sylvestre* extract improved blood glucose control compared with conventional preparations [122]. Overall, combining phytochemicals with advanced delivery systems represents a promising approach to enhance their therapeutic efficacy and clinical potential.

### 3.1. Diabetes and Osteoporosis

Diabetes is considered an important cause of secondary osteoporosis; people with diabetes have a significantly higher risk of fractures compared to the general population [123].

Diabetic osteoporosis (DO) is a metabolic bone disorder that is linked to chronic hyperglycemia. The prolonged hyperglycemic microenvironment in DO contributes to bone dysfunction in the metabolism as well as oxidative damage to progenitor cells [124].

Plants and their bioactive molecules play an important role in maintaining healthy bones, and targeting oxidative stress using medicinal plants represents a potential strategy to prevent osteoporosis. Vitamins such as D, K2, and biotin A have been shown to be very important in preventing diabetic osteoporosis [124]. Chen et al. showed that vitamin K2 decreases mitochondrial oxidative stress and metabolic disorders that are induced by a hyperglycemic environment [124]. Curcumin has been shown to control diabetic osteoporosis through increasing mitochondrial protection as well as decreasing oxidative stress and ROS-driven osteoblast apoptosis [124].

Data regarding the plants with known action against diabetes, including active parts and mechanisms, are summarized in Table 1 and Table 2.

### 3.2. Limitations and Future Research Directions

Despite the promising findings regarding the antidiabetic potential of various medicinal plants, several limitations remain holding back the research. For example, many studies rely on small sample sizes, single animal models, or short intervention periods, which may affect the reliability and broader applicability of their results. In addition, the lack of standardization in plant extracts and the incomplete characterization of active compounds make it more difficult to compare the findings across different studies.

Future research should prioritize well-designed, equally powered clinical trials to validate the efficacy and safety of these plant-based interventions. For example, standardized formulations and dosing regimens are needed, along with detailed identification of the active compounds and their mechanisms of action. Filling such gaps will help to better translate preclinical findings into practical and reliable interventions for diabetes management.

## 4. Conclusions

Plants have demonstrated significant potential as herbal medicines by serving as natural antioxidants and showing a high therapeutic value in the treatment of several diseases, including diabetes mellitus. While many studies report promising antidiabetic effects, most are limited because of small sample sizes, short experimental durations, or insufficient characterization of plant extracts. Addressing these limitations will be essential to developing safe, effective, and accessible herbal therapies.

When compared to conventional drugs, plants provide greater availability, lower cost, and better safety profiles. However, further research is required to identify additional plant species with antidiabetic potential, and to clarify precise molecular mechanisms, optimize the dosing, and explore the synergistic effects of combining different plant-derived molecules.

A deeper understanding of these interactions and the crosstalk between several affected cellular pathways is essential to fully identify the therapeutic benefits of medicinal plants in diabetes treatment. Integrating herbal medicines into modern diabetes care represents a promising frontier that requires continued research, standardization efforts, and collaborative approaches between traditional and modern medicine. Finally, as the global burden of diabetes continues to rise, the development of safe, effective, and accessible herbal therapies may provide a valuable complementary option for patients around the world, especially in resource-limited countries where conventional treatments may be less available or affordable.

## Figures and Tables

**Table 1:** A comprehensive summary of various plants known for their antidiabetic properties including the parts usually used for treatment procedures along with their respective mode of action and their corresponding therapeutic relevance.

Plant Name	Family	Used Plant Parts	Mode of Action	Ref.
*Achyranthes aspera*	Amaranthaceae	Seed, leaf	Inhibits the activities of glucosidase enzymes Reduces oxidative damage and increases the expression of the pancreatic insulin protein	[34,36]
*Allium sativum*	Amaryllidaceae	Whole plant	Inhibits the enzyme alpha glucosidase Increases insulin sensitivity	[40,41]
*Aloe vera*	Liliaceal	Whole plant	Inhibits glycation pathway Affects insulin secretion rate	[42,45]
*Amaranthus tricolor*	Amaranthaceae	Leaf and stem	Prevents oxidative stress in cells Stimulates anti-α-amylase, anti-α-glucosidase properties	[50,51]
*Anacardium occidentale*	Anacardiaceous	Leaf and stem	Improves hepatic and renal functions Enhances β-cell functions	[57]
*Annona squamosa*	Annonaceae	Roots, seeds, leaves, and fruits	Stimulates glucose uptake and the release of the insulin hormone	[61]
*Berberis vulgaris*	Berberidaceae	Fruit	Inhibits fructose-induced insulin resistance Downregulates the expression of aldose reductase Improves the sensitivity and the secretion of insulin Inhibits the release of glucagon Stimulates the proliferation of pancreatic β-cells and that of the GLP-1 hormone secretion which plays a role in insulin secretion Upregulates the expression of insulin receptor proteins Inhibits key enzymes contribution to glucose regulation	[63,64,66,67]
*Cinnamomum zeylanicum*	Lauraceae	Whole plant	Inhibits pancreatic α-amylase and α-glucosidase by stimulating the synthesis of glycogen and the metabolism of glucose Enhances GLUT-4 production and translocation	[70,71,73]
*Curcuma longa*	Zingiberaceae	Root	Improves the overall functions of b-cells Reduces the levels of metabolic parameters	[81,82]
*Gymnema sylvestre*	Asclepiadaceae	Leaves	Increases insulin secretion, inhibits intestinal glucose absorption, inhibits α-glucosidase enzyme	[84]
*Gynostemma pentaphylium*	Cucurbitaceae		Improves insulin sensitivity Increases the expression of GLUT4 Decreases the histological liver damage	[91,92]
*Momordica charantia*	Cucurbitaceae	Fruit	Controls glucose transportation Reduces gluconeogenic enzymes (such as glucose-6-phosphatase and fructosebiphosphatase) Increases the levels of intestinal Na^+^/glucose co-transporters, protectors of pancreatic islet β cells	[95,96,97]
*Nigella sativa*	Ranunculaceae	Whole plant	Blocks α-glucosidase and α-amylase digestive enzymes Reduces gluconeogenesis in the liver Inhibits the intestinal glucose transporters Increases the secretion of antioxidant enzymes Stimulates pancreatic-cell proliferation	[98,99,100,101]
*Ocimum sanctum*	Lamiaceae	Leaves	Increases the intra cellular calcium concentration of beta islet cells	[106]
*Punica granatum*	Lythraceae	Leave and flower	Increases the secretion of pancreatic β-cells Stimulates the mRNAs expression of IRS-1 and Akt genes Increases the activity of CAT enzymes and improves the health of pancreatic islets of Langerhans	[109,111]
*Trigonella foenum-graecum*	Fabaceae	Seeds and leaves	Overexpresses of GLUT2 mRNA Renews β-cell and promotes insulin secretion stimulation Inhibits lipid- and carbohydrate-hydrolyzing enzymes Stimulates translocation of GLUT4 to cell membrane	[114,115,116,117]

**Table 2:** Pharmacological data by study type.

Plant	Study Type	Model	Extract/Compound	Results	Ref
*Achyranthes aspera*	In vitro, in vivo	Rats	Ethanolic seed extracts	Reduced the blood glucose levels Inhibited the activities of α-amylase and α-glucosidase enzymes	[35,36]
*Allium sativum*	In vivo	Mice	Garlic polysaccharide	Reduced blood glucose; inhibited the enzyme α-glucosidase	[39,41]
*Aleo vera*	In vivo and in vitro and clinical	Rats	Aqueous crude extract (PBS-homogenized)	Increased serum insulin and decreased serum glucose	[45]
*Amaranthus tricolor*	In vivo and in vitro	Rats	70% ethanolic extract (in vivo). Water extract (in vitro)	Reduced the pancreatic malondialdehyde (MDA) levels and blood glucose. antidiabetic, anti-lipase, anti-α-amylase, anti-α-glucosidase, and anti-acetylcholinesterase	[50,51]
*Anacardium occidentale*	In vivo	Rats	Ethanolic extract	Reduced hyperglycemia	[55]
*Annona squamosa*	In vivo	Rats	Petroleum ether, ethyl acetate and alcoholic extracts	Blood glucose levels	[60]
*Berberis vulgaris*	In vivo and clinical	Rats, human	Water extract	Reduced blood glucose. downregulating aldose reductase expression and inhibiting its enzymatic activity	[65,66]
*Cinnamomum zeylanicum*	In vivo, clinical and in vitro	Rats, human	Water extract	Decrease plasma glucose and increase plasma insulin. GLUT-4 translocation	[72,75]
*Curcuma longa*	In vivo and clinical and in vitro	Mice, human	Ethanolic extract	Reduced reactive oxygen species (ROS) and fasting glucose levels	[79]
*Gymnema sylvestre*	In vivo	Rabbits	Ethanolic extract	Regeneration of pancreatic islet cells, increased insulin secretion, inhibition of intestinal glucose absorption, inhibition of α-glucosidase enzyme, enhancement of peripheral glucose metabolism	[85]
*Gynostemma pentaphyllum*	In vivo, in vitro and clinical	Mice, human	Water extract	Lowered plasma glucose	[91]
*Momordica charantia*	In vivo and vitro	Mice	Methanolic extract	Preserved the morphology and function of pancreatic islet β-cells, which are responsible for insulin secretion	[96]
*Nigella sativa*	In vivo	Rats	Essential oil extract	Protected the pancreatic islets	[103]
*Ocimum sanctum*	In vivo	Rats	Ethanolic extract	Enhanced the insulin secretion	[106]
*Punica granatum*	In vivo	Rat	Ethanolic extract	Resulted in reduced blood glucose levels	[110]
*Trigonella foenum-graecum* *ranatum*	In vitro	HepG2 cells	Water extract	Increased glucose uptake through upregulation of glucose transporter-2 (GLUT-2) mRNA levels	[116]

## Data Availability

Not applicable.

## References

[1] Antar S.A., Ashour N.A., Sharaky M., Khattab M., Ashour N.A., Zaid R.T., Roh E.J., Elkamhawy A., Al-Karmalawy A.A. (2023). Diabetes mellitus: Classification, mediators, and complications; A gate to identify potential targets for the development of new effective treatments. Biomed. Pharmacother..

[2] World Health Organization (2024). Diabetes.

[3] Shaikh A.A., Kolhatkar M.K., Sopane D.K., Thorve A.N. (2022). Review on: Diabetes mellitus is a disease. Int. J. Res. Pharm. Sci..

[4] Bastaki S. (2005). A review diabetes mellitus and its treatment. Int. J. Diabetes Metab..

[5] Dey R.K. (2023). Diabetes Mellitus: A comprehensive review of pathophysiology, management, and emerging therapeutic approaches. Diabetes Mellitus.

[6] Savan C., Viroja D., Kyada A. (2024). An updated review on diabetes mellitus: Exploring its etiology, pathophysiology, complications and treatment approach. IJCAAP.

[7] Lu X., Xie Q., Pan X., Zhang R., Zhang X., Peng G., Zhang Y., Shen S., Tong N. (2024). Type 2 diabetes mellitus in adults: Pathogenesis, prevention and therapy. Sig. Transduct. Target. Ther..

[8] Tegegne B.A., Adugna A., Yenet A., Yihunie Belay W., Yibeltal Y., Dagne A., Hibstu Teffera Z., Amare G.A., Abebaw D., Tewabe H. (2024). A critical review on diabetes mellitus type 1 and type 2 management approaches: From lifestyle modification to current and novel targets and therapeutic agents. Front. Endocrinol..

[9] Yameny A.A. (2024). Diabetes mellitus overview. J. Biosci. Appl. Res..

[10] Kaur S., Gadpayle D., Kumari A., Kaur G., Seen K., Bhardwaj R., Kumar A. (2025). Antidiabetic potential of underutilized crops: Nutritional, phytochemical insights, and prospects for diabetes management. Appl. Food Res..

[11] Chanda S., Ramachandra T.V. (2019). A review on some therapeutic aspects of phytochemicals present in medicinal plants. Int. J. Pharm. Life Sci..

[12] Nafiu M.O., Hamid A.A., Muritala H.F., Adeyemi S.B. (2023). Preparation, Standardization, and Quality Control of Medicinal Plants in Africa. Medicinal Plants: Ethnopharmacology, Phytochemistry and Therapeutic Applications.

[13] Whaley A.O., Whaley A.K., Kovaleva E.L., Frolova L.N., Orlova A.A., Luzhanin V.G., Flisyiuk E.V., Shigarova L.V., Pozharitskaya O.N., Shikov A.N. (2024). The Standardization of Officinal Medicinal Plants Used in the Eurasian Economic Union: Comparison with Other Pharmacopoeias. Pharm. Chem. J..

[14] Noviana E., Indrayanto G., Rohman A. (2022). Advances in Fingerprint Analysis for Standardization and Quality Control of Herbal Medicines. Front. Pharmacol..

[15] Forni C., Facchiano F., Bartoli M., Pieretti S., Facchiano A., D’Arcangelo D., Norelli S., Valle G., Nisini R., Beninati S. (2019). Beneficial Role of Phytochemicals on Oxidative Stress and Age-Related Diseases. Biomed. Res. Int..

[16] Al-Ishaq R.K., Overy A.J., Büsselberg D. (2020). Phytochemicals and Gastrointestinal Cancer: Cellular Mechanisms and Effects to Change Cancer Progression. Biomolecules.

[17] Begum V.S.M., Tariq N.P.M., Hemapriya J., Shariq K.M. (2022). Plants Secondary Metabolites as Medicines: A Review. IJZI.

[18] Enioutina E.Y., Salis E.R., Job K.M., Gubarev M.I., Krepkova L.V., Sherwin C.M.T. (2016). Herbal Medicines: Challenges in the modern world. Part 5. Status and current directions of complementary and alternative herbal medicine worldwide. Expert Rev. Clin. Pharmacol..

[19] Pérez-Flores J.G., García-Curiel L., Pérez-Escalante E., Contreras-López E., Aguilar-Lira G.Y., Ángel-Jijón C., González-Olivares L.G., Baena-Santillán E.S., Ocampo-Salinas I.O., Guerrero-Solano J.A. (2025). Plant Antimicrobial Compounds and Their Mechanisms of Action on Spoilage and Pathogenic Bacteria: A Bibliometric Study and Literature Review. Appl. Sci..

[20] Albahri G., Badran A., Hijazi A., Daou A., Baydoun E., Nasser M., Merah O. (2023). The therapeutic wound healing bioactivities of various medicinal plants. Life.

[21] Sundaram M.K., Khan M.A., Alalami U., Somvanshi P., Bhardwaj T., Pramodh S., Raina R., Shekfeh Z., Haque S., Hussain A. (2020). Phytochemicals induce apoptosis by modulation of nitric oxide signaling pathway in cervical cancer cells. Eur. Rev. Med. Pharmacol. Sci..

[22] Lin R., Hu X., Chen S., Shi Q., Chen H. (2020). Naringin induces endoplasmic reticulum stress-mediated apoptosis, inhibits β-catenin pathway and arrests cell cycle in cervical cancer cells. Acta Biochim. Pol..

[23] Mahmoud V.L., Shayesteh R., Foong Yun Loh T.K., Chan S.W., Sethi G., Burgess K., Lee S.H., Wong W.F., Looi C.Y. (2024). Comprehensive Review of Opportunities and Challenges of Ethnomedicinal Plants for Managing Type 2 Diabetes. Heliyon.

[24] Hui H., Tang G., Go V. (2009). Hypoglycemic Herbs and Their Action Mechanisms. Chin. Med..

[25] Kooti W., Farokhipour M., Asadzadeh Z., Ashtary-Larky D., Asadi-Samani M. (2016). The Role of Medicinal Plants in the Treatment of Diabetes: A Systematic Review. Electron. Physician.

[26] Salleh N.H., Zulkipli I.N., Mohd Yasin H., Ja’afar F., Ahmad N., Wan Ahmad W.A.N., Ahmad S.R. (2021). Systematic Review of Medicinal Plants Used for Treatment of Diabetes in Human Clinical Trials: An ASEAN Perspective. Evid.-Based Complement. Altern. Med..

[27] Balkrishna A., Sharma N., Srivastava D., Kukreti A., Srivastava S., Arya V. (2024). Exploring the Safety, Efficacy, and Bioactivity of Herbal Medicines: Bridging Traditional Wisdom and Modern Science in Healthcare. Future Integr. Med..

[28] Mourya R., Sharma R. (2025). Interaction Between Anti-Diabetic Drugs and Herbs: A Review. Pharmacogn. Res..

[29] Dash J.R., Kar B., Pattnaik G. (2020). Pharmacological Interaction Between Anti-Diabetic Drugs and Herbs: An Overview of Mechanism of Action and Clinical Implication. Plant Arch..

[30] Li X., Geng-Ji J.-J., Quan Y.-Y., Qi L.-M., Sun Q., Huang Q., Jiang H.-M., Sun Z.-J., Liu H.-M., Xie X. (2022). Role of Potential Bioactive Metabolites from Traditional Chinese Medicine for Type 2 Diabetes Mellitus: An Overview. Front. Pharmacol..

[31] Chattopadhyay K., Wang H., Kaur J., Nalbant G., Almaqhawi A., Kundakci B., Panniyammakal J., Heinrich M., Lewis S.A., Greenfield S.M. (2022). Effectiveness and Safety of Ayurvedic Medicines in Type 2 Diabetes Mellitus Management: A Systematic Review and Meta-Analysis. Front. Pharmacol..

[32] Abu-Odeh A.M., Talib W.H. (2021). Middle East Medicinal Plants in the Treatment of Diabetes: A Review. Molecules.

[33] Zanzabil K.Z., Hossain M.S., Hasan M.K. (2023). Diabetes mellitus management: An extensive review of 37 medicinal plants. Diabetology.

[34] Asiago O.H., Reddy K.S. (2023). Protective effect of achyranthes aspera against high fat diet and streptozotocin induced diabetes in rats [Adventure]. J. Chem. Health Risks.

[35] Vijayaraj R., Naresh Kumar K., Mani P., Senthil J., Jayaseelan T., Dinesh Kumar G. (2016). Hypoglycemic and antioxidant activity of *Achyranthes aspera* seed extract and its effect on streptozotocin induced diabetic rats. Int. J. Biol. Pharm. Res..

[36] Priyamvada P.M., Mishra P., Sha A., Mohapatra A.K. (2021). Evaluation of antidiabetic and antioxidant activities of *Achyranthes aspera* leaf extracts: An in vitro study. Int. J. Pharm. Life Sci..

[37] Njideka B.E., Theophilus A.E.N., Ugochukwu N.T. (2019). Use of *Achyranthes aspera* Linn Tea as antidiabetic and hypolipidemic herbal tea. Int. J. Health Sci. Res..

[38] Sanie-Jahromi F., Zia Z., Afarid M. (2023). A review on the effect of garlic on diabetes, BDNF, and VEGF as a potential treatment for diabetic retinopathy. Chin. Med..

[39] Xie C., Gao W., Li X., Luo S., Wu D., Chye F.Y. (2023). Garlic (*Allium sativum* L.) polysaccharide ameliorates type 2 diabetes mellitus (T2DM) via the regulation of hepatic glycogen metabolism. NFS J..

[40] Najafi N., Masoumi S.J. (2018). The Effect of garlic (*allium sativum*) supplementation in patients with type 2 diabetes mellitus: A systematic review. Int. J. Nutr. Sci..

[41] Abdullah H., Miladiyah I., Nurdiyanto H., Miladiyah I., Jamil N.A. (2023). Garlic (*Allium sativum* L.) Efficacy as an adjuvant therapy for type 2 diabetes mellitus: A scoping review. Proceedings of the 3rd International Conference on Cardiovascular Diseases (ICCvD 2021).

[42] Harshali, Thakur P., Mukherjee G. (2021). Aloe Vera as an Antidiabetic and Wound Healing Agent for Diabetic Patients. JPRI.

[43] Zhang Y., Liu W., Liu D., Zhao T., Tian H. (2016). Efficacy of Aloe Vera supplementation on prediabetes and early non-treated diabetic patients: A Systematic review and meta-analysis of randomized controlled trials. Nutrients.

[44] Budiastutik I., Subagio H.W., Kartasurya M.I., Widjanarko B., Kartini A., Soegiyanto S., Suhartono S.S. (2022). The effect of aloe vera on fasting blood glucose levels in pre-diabetes and type 2 diabetes mellitus: A systematic review and meta-analysis. J. Pharm. Pharmacogn. Res..

[45] Abo-Youssef A.M.H., Messiha B.A.S. (2013). Beneficial effects of aloe vera in treatment of diabetes: Comparative in vivo and in vitro studies. Bull. Fac. Pharm. Cairo Univ..

[46] Fallah Huseini H., Kianbakht S., Hajiaghaee R., Afkhami-Ardekani M., Bonakdaran A., Hashem Dabaghian F. (2012). *Aloe vera* Leaf Gel in Treatment of Advanced Type 2 Diabetes Mellitus Needing Insulin Therapy: A Randomized Double-Blind Placebo-Controlled Clinical Trial. J. Med. Plants.

[47] Rahman A.H.M.M., Iffat Ara Gulshana M. (2014). Taxonomy and medicinal uses on amaranthaceae family of rajshahi, Bangladesh. AEES.

[48] Aneja S., Vats M., Aggarwal S., Sardana S. (2013). Phytochemistry and hepatoprotective activity of aqueous extract of *Amaranthus tricolor* Linn. Roots. J. Ayurveda Integr. Med..

[49] Rahmatullah M., Hosain M., Rahman S., Rahman S., Akter M., Rahman F., Rehana F., Munmun M., Kalpana M. (2013). antihyperglycemic and antinociceptive activity evaluation of methanolic extract of whole plant of Amaranthus Tricolor L. (Amaranthaceae). Afr. J. Tradit. Complement. Altern. Med..

[50] Rahma K., Nurcahyanti O. (2021). Therapeutic effect of red spinach (*Amaranthus tricolor* L.) extract on pancreatic MDA levels rats (Rattus norvegicus) exposed to MLD-STZ. J. Biomed. Transl. Res..

[51] Yang Y.-C., Mong M.-C., Wu W.-T., Wang Z.-H., Yin M.-C. (2020). Phytochemical profiles and anti-diabetic benefits of two edible amaranthus species. CyTA J. Food.

[52] Ajao F.O., Iyedupe M.O., Akanmu O., Kalejaiye N.O., Adegoke A.L., Adeniji L.A. (2023). Anti-oxidative, anti-inflammatory and anti-apoptotic efficacy of *Anacardium occidentale* leaf extract in diabetic rats. Int. J. Diabetes Clin. Res..

[53] Jaiswal Y.S., Tatke P.A., Gabhe S.Y., Vaidya A.B. (2017). Antidiabetic activity of extracts of *Anacardium occidentale* Linn. leaves on n-streptozotocin diabetic rats. J. Tradi. Complement. Med..

[54] Ponce-Mora A., Gimeno-Mallench L., Lavandera J.L., Giebelhaus R.T., Domenech-Bendaña A., Locascio A., Gutierrez-Rojas I., Sauro S., De La Mata P., Nam S.L. (2025). Systematic Characterization of Antioxidant Shielding Capacity Against Oxidative Stress of Aerial Part Extracts of Anacardium occidentale. Antioxidants.

[55] Abdullahi S., Olatunji G.A. (2010). Antidiabetic activity of *Anacardium occidentale* in alloxan—Diabetic rats. Jnl Sci. Tech..

[56] Olatunji L.A., Okwusidi J.I., Soladoye A.O. (2005). Antidiabetic effect of *Anacardium occidentale*. Stem-bark in fructose-diabetic rats. Pharm. Biol..

[57] Ukwenya V.O., Alese M.O., Ogunlade B., Folorunso I.M., Omotuyi O.I. (2022). Anacardium occidentale leaves extract and riboceine mitigate hyperglycemia through anti-oxidative effects and modulation of some selected genes associated with diabetes. J. Diabetes Metab. Disord..

[58] Sharma C.P., Singh A., Prasad R.K., Mishra D.K., Singh A.K., Yadav S. (2024). Antidiabetic and antioxidant activity of *Annona squamosa* bark using successive solvent extraction method. J. Complement. Herb. Res..

[59] Almalki G., Alothman N., Mohamed G., Akeel M., El-Beltagy A.E.-F.B.M., Salem E.T. (2024). Modulatory role of *Annona squamosa* extract against streptozotocin-induced diabetic nephropathy in male rats. Egypt. J. Basic Appl. Sci..

[60] Sharma A., Chand T., Khardiya M., Yadav K.C., Mangal R., Sharma A.K. (2013). Antidiabetic and antihyperlipidemic activity of *Annona squamosa* fruit peel in streptozotocin induced diabetic rats. Int. J. Pharm. Sci. Res..

[61] Gupta R.K., Kesari A.N., Watal G., Murthy P.S., Chandra R., Maithal K., Tandon V. (2005). Hypoglycaemic and antidiabetic effect of aqueous extract of leaves of *Annona squamosa* (L.) in experimental animals. Curr. Sci..

[62] Kaleem M., Asif M., Ahmed Q.U., Bano B. (2006). Antidiabetic and antioxidant activity of Annona squamosa extract in streptozotocin-induced diabetic rats. Singap. Med. J..

[63] Muszalska A., Wiecanowska J. (2024). *Berberis vulgaris*: A natural source of berberine for addressing contemporary health concerns. Herba. Pol..

[64] Pang B., Zhao L.-H., Zhou Q., Zhao T.-Y., Wang H., Gu C.-J., Tong X.-L. (2015). Application of berberine on treating type 2 diabetes mellitus. Int. J. Endocrinol..

[65] Meliani N., Dib M.E.A., Allali H., Tabti B. (2011). Hypoglycaemic effect of *Berberis vulgaris* L. in normal and streptozotocin-induced diabetic rats. Asian Pac. J. Trop. Biomed..

[66] Shidfar F., Ebrahimi S.S., Hosseini S., Heydari I., Shidfar S., Hajhassani G. (2012). The effects of *Berberis vulgaris* fruit extract on serum lipoproteins, apoB, apoA-I, homocysteine, glycemic control and total antioxidant capacity in type 2 diabetic patients. Iran J. Pharm. Res..

[67] Belwal T., Bisht A., Devkota H.P., Ullah H., Khan H., Pandey A., Bhatt I.D., Echeverría J. (2020). Phytopharmacology and Clinical Updates of *Berberis* Species Against Diabetes and Other Metabolic Diseases. Front. Pharmacol..

[68] Munguia-Nolan J.E., García-Puga J.A., Robles-Zepeda R.E., Quintana-Zavala M.O., Díaz-Zavala R.G., Rendón-Domínguez I.P. (2024). Efectos de *Cinnamomum zeylanicum* en Niveles Glucémicos en Pacientes con Diabetes Tipo 2: Ensayo Clínico Aleatorizado. Enf. Global.

[69] Senevirathne B.S., Jayasinghe M.A., Pavalakumar D., Siriwardhana C.G. (2022). Ceylon cinnamon: A versatile ingredient for futuristic diabetes management. J. Future Foods.

[70] Ranasinghe P., Jayawardana R., Galappaththy P., Constantine G.R., De Vas Gunawardana N., Katulanda P. (2012). Efficacy and safety of ‘true’ cinnamon (*Cinnamomum zeylanicum*) as a pharmaceutical agent in diabetes: A Systemic review and meta-analysis. Diabet. Med..

[71] Shihabudeen H.M.S., Priscilla D.H., Thirumurugan K. (2011). Cinnamon extract inhibits α-glucosidase activity and dampens postprandial glucose excursion in diabetic rats. Nutr. Metab..

[72] Medagama A.B. (2015). The glycaemic outcomes of Cinnamon, a review of the experimental evidence and clinical trials. Nutr. J..

[73] Shen Y., Fukushima M., Ito Y., Muraki E., Hosono T., Seki T., Ariga T. (2010). Verification of the antidiabetic effects of Cinnamon (*Cinnamomum zeylanicum*) using insulin-uncontrolled type 1 diabetic rats and cultured adipocytes. Biosci. Biotechnol. Biochem..

[74] Yaghmoor S.S., Khoja S.M. (2010). Effect of Cinnamon on plasma glucose concentration and the regulation of phosphofructo-1-kinase activity from the liver and small intestine of streptozotocin induced diabetic rats. J. Biol. Sci..

[75] Taher M., Abdul Majid F.A., Sarmidi M.R. (2006). A proanthocyanidin from *Cinnamomum zeylanicum* stimulates phosphorylation of insulin receptor in 3T3–L1 adipocytes. J. Teknol..

[76] Marton L.T., Pescinini-e-Salzedas L.M., Camargo M.E.C., Barbalho S.M., Haber J.F.D.S., Sinatora R.V., Detregiachi C.R.P., Girio R.J.S., Buchaim D.V., Cincotto Dos Santos Bueno P. (2021). The effects of curcumin on diabetes mellitus: A systematic review. Front. Endocrinol..

[77] Zhang D., Fu M., Gao S.-H., Liu J.-L. (2013). Curcumin and diabetes: A systematic review. Evid. Based Complement. Altern. Med..

[78] Rivera-Mancía S., Trujillo J., Chaverri J.P. (2018). Utility of curcumin for the treatment of diabetes mellitus: Evidence from preclinical and clinical studies. J. Nutr. Intermed. Metab..

[79] Chuengsamarn S., Rattanamongkolgul S., Luechapudiporn R., Phisalaphong C., Jirawatnotai S. (2012). Curcumin extract for prevention of type 2 diabetes. Diabetes Care.

[80] Pathomwichaiwat T., Jinatongthai P., Prommasut N., Ampornwong K., Rattanavipanon W., Nathisuwan S., Thakkinstian A. (2023). Effects of turmeric (curcuma longa) supplementation on glucose metabolism in diabetes mellitus and metabolic syndrome: An umbrella review and updated meta-analysis. PLoS ONE.

[81] Lu W., Khatibi F., Shahidi F., Khorsandi K., Hossienzadeh R., Asma G., Balck V. (2022). An update on molecular mechanisms of curcumin effect on diabetes. Fundam. Clin. Pharmacol..

[82] Abbas W., Khan R.A., Baig M.T., Shaikh S.A., Kumar A. (2021). Role of Curcuma Longa in Type 2 Diabetes and Its Associated Complications. JPRI.

[83] Kanetkar P., Singhal R., Kamat M. (2007). *Gymnema sylvestre*: A memoir. J. Clin. Biochem. Nutr..

[84] Muzaffar H., Qamar I., Bashir M., Jabeen F., Irfan S., Anwar H. (2023). *Gymnema sylvestre* Supplementation Restores Normoglycemia, Corrects Dyslipidemia, and Transcriptionally Modulates Pancreatic and Hepatic Gene Expression in Alloxan-Induced Hyperglycemic Rats. Metabolites.

[85] Yadav D., Kwak M., Jin J.-O. (2019). Clinical applications of *Gymnema sylvestre* against type 2 diabetes mellitus and its associated abnormalities. Prog. Nutr..

[86] Kashif M., Nasir A., Gulzaman, Rafique M.K., Abbas M., Ur Rehman A., Riaz M., Rasool G., Mtewa A.G. (2023). Unlocking the anti-diabetic potential of Gymnema syvestre, Trigonella foenum-graecum, and their combination thereof: An in vivo evaluation. Food Sci. Nutr..

[87] Gaonkar V.P., Hullatti K. (2020). Indian Traditional medicinal plants as a source of potent Anti-diabetic agents: A Review. J. Diabetes Metab. Disord..

[88] Huyen V.T.T., Phan D.V., Thang P., Ky P.T., Hoa N.K., Ostenson C.G. (2012). Antidiabetic effects of add-on *Gynostemma pentaphyllum* extract therapy with sulfonylureas in type 2 diabetic patients. Evid. Based Complement. Altern. Med..

[89] Li X., Liu L., Wei S. (2025). *Gynostemma pentaphyllum*: A review on its traditional uses, phytochemistry and pharmacology. J. Funct. Foods.

[90] Song M., Tan D., Li B., Wang Y., Shi L. (2022). Gypenoside ameliorates insulin resistance and hyperglycemia via the AMPK-mediated signaling pathways in the liver of type 2 diabetes mellitus mice. Food Sci. Hum. Wellness.

[91] Huyen V.T.T., Phan D.V., Thang P., Hoa N.K., Östenson C.G. (2013). *Gynostemma pentaphyllum* tea improves insulin sensitivity in type 2 diabetic patients. J. Nutr. Metab..

[92] Xie J.-B., Xie P., Guo M., Li F.-F., Xiao M.-Y., Qi Y.-S., Pei W.-J., Luo H.-T., Gu Y.-L., Piao X.-L. (2023). Protective effect of heat-processed *Gynostemma pentaphyllum* on high fat diet-induced glucose metabolic disorders mice. Front. Pharmacol..

[93] Joseph B., Jini D. (2013). Antidiabetic effects of *Momordica charantia* (bitter melon) and its medicinal potency. Asian Pac. J. Trop. Dis..

[94] Leung L., Birtwhistle R., Kotecha J., Hannah S., Cuthbertson S. (2009). Anti-diabetic and hypoglycaemic effects of *Momordica charantia* (Bitter Melon): A mini review. Br. J. Nutr..

[95] Richter E., Geetha T., Burnett D., Broderick T.L., Babu J.R. (2023). The effects of *Momordica charantia* on Type 2 Diabetes Mellitus and Alzheimer’s Disease. Int. J. Mol. Sci..

[96] Garau C., Cummings E., Phoenix D.A., Singh J. (2003). Beneficial effect and mechanism of action of *Momordica charantia* in the treatment of diabetes mellitus: A mini review. Int. J. Diabetes Metab..

[97] Oyelere S.F., Ajayi O.H., Ayoade T.E., Santana Pereira G.B., Dayo Owoyemi B.C., Ilesanmi A.O., Akinyemi O.A. (2022). A detailed review on the phytochemical profiles and anti-Diabetic mechanisms of *Momordica charantia*. Heliyon.

[98] Shaukat A., Zaidi A., Anwar H., Kizilbash N. (2023). Mechanism of the antidiabetic action of *Nigella sativa* and Thymoquinone: A review. Front. Nutr..

[99] Maideen N.M.P. (2021). Antidiabetic activity of nigella sativa (black seeds) and its active constituent (thymoquinone): A review of human and experimental animal studies. Chonnam Med. J..

[100] Mashayekhi-Sardoo H., Sepahi S., Baradaran Rahimi V., Askari V.R. (2024). Application of *Nigella sativa* as a functional food in diabetes and related complications: Insights on molecular, cellular, and metabolic effects. J. Funct. Foods.

[101] Hamdan A., Haji Idrus R., Mokhtar M.H. (2019). Effects of nigella sativa on type-2 diabetes mellitus: A systematic review. Int. J. Environ. Res. Public Health.

[102] El-Aarag B., Hussein W., Ibrahim W., Zahran M. (2017). Thymoquinone improves anti-diabetic activity of metformin in streptozotocin-induced diabetic male rats. J. Diabetes Metab..

[103] Afaf Jamal Ali Hmza E., Omar A., Adnan A., Osman M.T. (2013). *Nigella sativa* oil has significant repairing ability of damaged pancreatic tissue occurs in induced type 1 diabetes mellitus. Glob. J. Pharmacol..

[104] Choo T.-M. (2023). *Nigella sativa* tea mitigates type-2 diabetes and edema: A case report. Adv. Tradit. Med..

[105] Hannan J.M.A., Marenah L., Ali L., Rokeya B., Flatt P.R., Abdel-Wahab Y.H.A. (2006). *Ocimum sanctum* leaf extracts stimulate insulin secretion from perfused pancreas, isolated islets and clonal pancreatic β-Cells. J. Endocrinol..

[106] Abhilash Rao S., Vijay Y., Deepthi T., Sri Lakshmi C., Vibha Rani S., Swetha Rani B., Bhuvaneswara Reddy Y., Ram Swaroop P., Sai Laxmi V., Nikhil Chakravarthy K. (2013). Anti-diabetic effect of ethanolic extract of leaves of *ocimum sanctum* in alloxan induced diabetes in rats. Int. J. Basic Clin. Pharmacol..

[107] Suanarunsawat T., Anantasomboon G., Piewbang C. (2016). Anti-diabetic and anti-oxidative activity of fixed oil extracted from *Ocimum sanctum* L. leaves in diabetic rats. Exp. Ther. Med..

[108] Cheurfa M., Achouche M., Azouzi A., Abdalbasit M.A. (2020). Antioxidant and anti-diabetic activity of pomegranate (*Punica granatum* L.) leaves extracts. Foods Raw Maters..

[109] Gharib E., Kouhsari S.M. (2019). Study of the antidiabetic activity of *Punica granatum* L. fruits aqueous extract on the alloxan-diabetic wistar rats. Iran. J. Pharm. Res..

[110] Tang D., Liu L., Ajiakber D., Ye J., Xu J., Xin X., Aisa H.A. (2018). Anti-diabetic Effect of *Punica granatum* Flower Polyphenols Extract in Type 2 Diabetic Rats: Activation of Akt/GSK-3β and Inhibition of IRE1α-XBP1 Pathways. Front. Endocrinol..

[111] Mabrouk Gabr N. (2017). Effects of pomegranate (*Punica granatum* L.) fresh juice and peel extract on diabetic male albino rats. AMJ.

[112] Laila O., Murtaza I., Muzamil S., Imtiyaz Ali S., Abid Ali S., Ahamad Paray B., Gulnaz A., Vladulescu C., Mansoor S. (2023). Enhancement of nutraceutical and anti-diabetic potential of fenugreek (*Trigonella foenum-graecum*). Sprouts with natural elicitors. Saudi Pharm. J..

[113] Baset M.E., Ali T.I., Elshamy H., El Sadek A.M., Sami D., Badawy M., Abou-Zekry S., Heiba H., Saadeldin M., Abdellatif A. (2020). Anti-Diabetic Effects of Fenugreek (*Trigonella foenum-graecum*): A Comparison between Oral and Intraperitoneal Administration—An Animal Study. Int. J. Funct. Nutr..

[114] Kumar A., Aswal S., Chauhan A., Semwal R.B., Kumar A., Semwal D.K. (2019). Ethnomedicinal investigation of medicinal plants of Chakrata region (Uttarakhand) used in the traditional medicine for diabetes by Jaunsari tribe. Nat. Prod. Bioprospect..

[115] Ota A., Ulrih N.P. (2017). An overview of herbal products and secondary metabolites used for management of type two diabetes. Front. Pharmacol..

[116] Haxhiraj M., White K., Terry C. (2024). The role of fenugreek in the management of type 2 diabetes. Int. J. Mol. Sci..

[117] Sarker D.K., Ray P., Dutta A.K., Rouf R., Uddin S.J. (2024). Antidiabetic potential of fenugreek (*Trigonella foenum-graecum*): A magic herb for diabetes mellitus. Food Sci. Nutr..

[118] Patel J., Patel R., Khambholja K., Patel N. (2008). An overview of phytosomes as an advanced herbal drug delivery system. Asian J. Pharm. Sci..

[119] Epriliati I., Ginjom I.R., Rao V. (2012). Bioavailability of Phytochemicals. Phytochemicals—A Global Perspective of Their Role in Nutrition and Health.

[120] Lv Y., Li W., Liao W., Jiang H., Liu Y., Cao J., Lu W., Feng Y. (2024). Nano-drug delivery systems based on natural products. Int. J. Nanomed..

[121] Yallapu M.M., Jaggi M., Chauhan S.C. (2012). Curcumin nanoformulations: A future nanomedicine for cancer. Drug Discov. Today.

[122] Poorani V., Selvakumar K., Venkat Kumar G. (2020). Improving bioavailability of phytochemicals through niosomes. J. Drug Deliv. Ther..

[123] Prasad T.N., Arjunan D., Pal R., Bhadada S.K. (2023). Diabetes and Osteoporosis. Indian J. Orthop..

[124] Mohammad O.H., Yang S., Ji W., Ma H., Tao R. (2025). Curcumin preserves bone health compromised by diabetes by inhibiting osteoporosis through regulation of the SIRT3/FoxO3a signalling pathway. Sci. Rep..

